# A coupled-oscillator model of olfactory bulb gamma oscillations

**DOI:** 10.1371/journal.pcbi.1005760

**Published:** 2017-11-15

**Authors:** Guoshi Li, Thomas A. Cleland

**Affiliations:** Dept. Psychology, Cornell University, Ithaca, NY United States of America; University of Pittsburgh, UNITED STATES

## Abstract

The olfactory bulb transforms not only the information content of the primary sensory representation, but also its underlying coding metric. High-variance, slow-timescale primary odor representations are transformed by bulbar circuitry into secondary representations based on principal neuron spike patterns that are tightly regulated in time. This emergent fast timescale for signaling is reflected in gamma-band local field potentials, presumably serving to efficiently integrate olfactory sensory information into the temporally regulated information networks of the central nervous system. To understand this transformation and its integration with interareal coordination mechanisms requires that we understand its fundamental dynamical principles. Using a biophysically explicit, multiscale model of olfactory bulb circuitry, we here demonstrate that an inhibition-coupled intrinsic oscillator framework, pyramidal resonance interneuron network gamma (PRING), best captures the diversity of physiological properties exhibited by the olfactory bulb. Most importantly, these properties include global zero-phase synchronization in the gamma band, the phase-restriction of informative spikes in principal neurons with respect to this common clock, and the robustness of this synchronous oscillatory regime to multiple challenging conditions observed in the biological system. These conditions include substantial heterogeneities in afferent activation levels and excitatory synaptic weights, high levels of uncorrelated background activity among principal neurons, and spike frequencies in both principal neurons and interneurons that are irregular in time and much lower than the gamma frequency. This coupled cellular oscillator architecture permits stable and replicable ensemble responses to diverse sensory stimuli under various external conditions as well as to changes in network parameters arising from learning-dependent synaptic plasticity.

## Author summary

The mammalian olfactory bulb responds to odor stimulation by generating fast oscillations in its electrical field potential. Such oscillations are indications that a substantial number of principal neurons in the olfactory bulb are coordinating their activities in time, which often means that their action potentials are synchronized, or partly synchronized, such that the pattern of small differences in their spike times contains olfactory sensory information. We are interested in the mechanisms by which olfactory bulb circuitry can transform sensory information from the temporally unsophisticated spike rates of primary sensory neurons into this sophisticated cortical format. We present a biophysically explicit, multiscale dynamical model of the olfactory bulb network that generates these oscillations. The elements of this model are designed to adhere to experimental findings from individual neurons, membrane currents, and synapses as well as the functional network. Together, these elements generate gamma oscillations exhibiting the full range of properties of those in the biological system. We show that these dynamics arise from an inhibition-coupled oscillator framework, a type of dynamical system with some established mathematical properties. This finding enables us to understand how the olfactory system translates sensory information for distribution in the central nervous system, and how different areas of the brain can mechanistically coordinate with one another so as to regulate the flow of sensory information to appropriate target structures.

## Introduction

The mammalian main olfactory bulb (OB) plays a central role in processing and relaying olfactory information from the primary sensory epithelium to subcortical and cortical areas [1]. This processing transforms the information content of the primary representation, but also has been proposed to transform the underlying physical metric by which this information is encoded, from rate-coded population activity organized on a respiration timescale to a spike timing-based representation aligned to a faster timescale that is determined by the intrinsic dynamics of cortical neural ensembles [2]. Odor stimulus-evoked activation of the OB generates fast, gamma-band (30–80 Hz) local field potential (LFP) oscillations that are thought to be largely synchronous across the extent of the OB [3]. Such oscillations reflect the tightly constrained synchronization of a large neural assembly, which in the OB (and its arthropod analogues) has long been believed to play some role in the encoding and processing of olfactory information [4–14].

**Fig 1:**
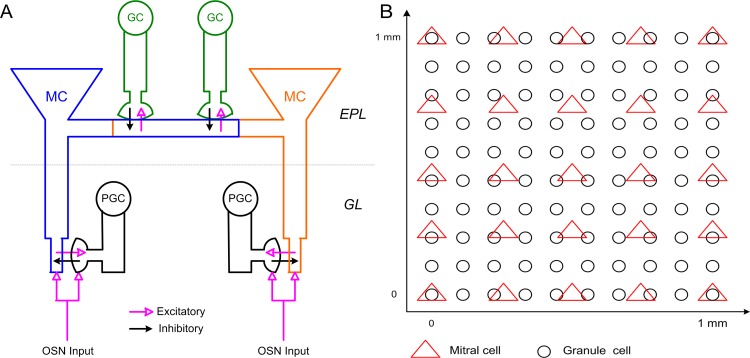
Schematic representation of OB network connectivity and model structure. ***A*:** Schematic representation of dendrodendritic synaptic connectivity among MCs, PGCs, and GCs. Reciprocal dendrodendritic synaptic connections exist between the MC tuft and PGC spines, and between the MC lateral dendrite and GC spines. GL: glomerular layer; EPL: external plexiform layer. ***B*:** Spatial localization of MCs and GCs across the two-dimensional toroidal surface of the model OB (1 mm × 1 mm).

It is generally accepted that OB gamma oscillations are intrinsic, and mediated by a fast negative feedback loop formed between principal output neurons (mitral and projecting tufted cells; MCs) and a class of inhibitory GABAergic interneurons (granule cells; GCs), interacting via dendrodendritic synapses in the external plexiform layer (EPL) of the OB (Fig 1A; [15–25]). However, the underlying mechanisms generating these oscillations remain elusive. Several different dynamical architectures have been proposed or assumed to mediate OB gamma oscillogenesis. First, a pyramidal-interneuron network gamma (PING) mechanism is often assumed [22, 26], inspired by the anatomical predominance of the excitatory-inhibitory reciprocal synapses that constitute the EPL network. Early theoretical modeling of OB network dynamics also was based on this anatomical architecture [27]. However, PING networks do not incorporate cellular resonance properties, such as the intrinsic subthreshold oscillations (STOs) of MCs [28]. Second, an interneuron network gamma (ING) mechanism has been theoretically proposed [29, 30]; however, this mechanism relies on inhibitory interactions among granule cells, which were intimated by early EEG work [16] and by the discovery of GABAergic synaptic inputs onto granule cells [31] but since have been ruled out. The PING and ING architectures have been reviewed by [32]. Third, OB network oscillations have been proposed to be driven directly by the intrinsic subthreshold dynamics of MCs [33]. This model highlighted the dynamical capacities of intrinsic MC subthreshold oscillations (STOs; [28]) and resolved some limitations of the PING architecture regarding observed OB dynamics (e.g., it permitted stable gamma oscillation frequency in the presence of fluctuating afferent drive). However, this model required substantially higher-frequency MC STOs than have been experimentally described, and also was not clearly compatible with the sparse spiking behavior of GCs [34]. Fourth, a hybrid network based on inhibition-coupled intrinsic cellular oscillators has been proposed [35], in which the intrinsic STOs of MCs are transiently coupled during afferent activation into a coherent oscillatory network [36] paced by GC-mediated inhibitory synaptic inputs that periodically reset the slower MC STOs. (Pulsed inhibitory inputs, including shunting inhibition, have been demonstrated to effectively reset MC STOs [28, 37–39]). During these active epochs, the network dynamics exhibit key PING-like properties (e.g., the population oscillation frequency depends on the decay time constant of the GABA(A) receptor conductance), but they also retain a dependence on the slower STO dynamics of mitral cells even when the STO frequency itself is superseded by the network oscillation. This dynamical mechanism, pyramidal resonance interneuron network gamma (PRING), is consistent with a broad range of experimental data and is modeled here.

It is important to clearly understand the specific dynamical mechanisms underlying OB field oscillations, for several reasons. These oscillations are likely to reflect the re-encoding of afferent odor information into timing-based representations for distribution to multiple postbulbar cortical and subcortical structures [40]. Therefore, in order to understand the formation and information content of these secondary representations, the dynamics of their creation must be clear. Moreover, ascending inputs from the anterior olfactory nucleus and piriform cortex, among other structures, must be integrated into this dynamical framework. Piriform cortical inputs, in particular, are understood to alter bulbar dynamics, transiently transforming the OB’s intrinsic gamma oscillations into slower beta-band oscillations coherent with those of the piriform cortex [13, 41–44]. To understand how these ascending inputs are integrated into the secondary odor representation requires a correct, mechanistic model of bulbar gamma oscillogenesis and its subversion by piriform cortical activity. Finally, field potential oscillations at many different characteristic frequencies are found all over the brain, often interacting within particular neural structures [45] and potentially serving to select and route specific information between coherently activated brain regions [46]. Elucidation of the detailed mechanics of oscillations and their transitions in the OB and its associated networks hence also will pertain to broader questions surrounding interareal communication mechanisms in the brain.

To address this question, we developed a conductance-based, dynamically detailed biophysical model of the OB network. The present model is based on our earlier two-layer model of cholinergic neuromodulation in the OB [25], but embeds these glomerular layer and intercolumnar EPL computations within an explicit spatial framework. The results from this model favor the PRING mechanism described above [35], and demonstrate that this inhibition-coupled cellular oscillator architecture supports the diverse phenomena observed in OB neurophysiological recordings. These phenomena include (1) patterned spiking activity in MCs and GCs that both is broadly heterogeneous and occurs at lower frequencies than the population rhythm, (2) tolerance to a wide range of afferent MC excitation levels, which is important for mediating the representation of different odor qualities, (3) tolerance for substantial changes in MC-GC synaptic weights, which underlie intrinsic odor learning within the OB [47–49], (4) the broad coherence of gamma-band oscillations across a physically extensive network despite the incoherent activity of some neurons within that network, (5) the phase-constraining of spikes within each cycle of the gamma oscillation [10, 13], and (6) the persistence of LFP gamma oscillations at consistent frequencies despite sparse network connectivity (connection probability *p* = 0.3 between MCs and GCs) and sharply heterogeneous afferent activation levels. The explicit, multiscale nature of this dynamical model further enables the elaboration, explanation, and experimental testing of the underlying mechanistic details that may underlie these observed physiological phenomena.

## Results

### Odor stimulation induces broadly coherent gamma oscillations across the OB network

**Fig 2:**
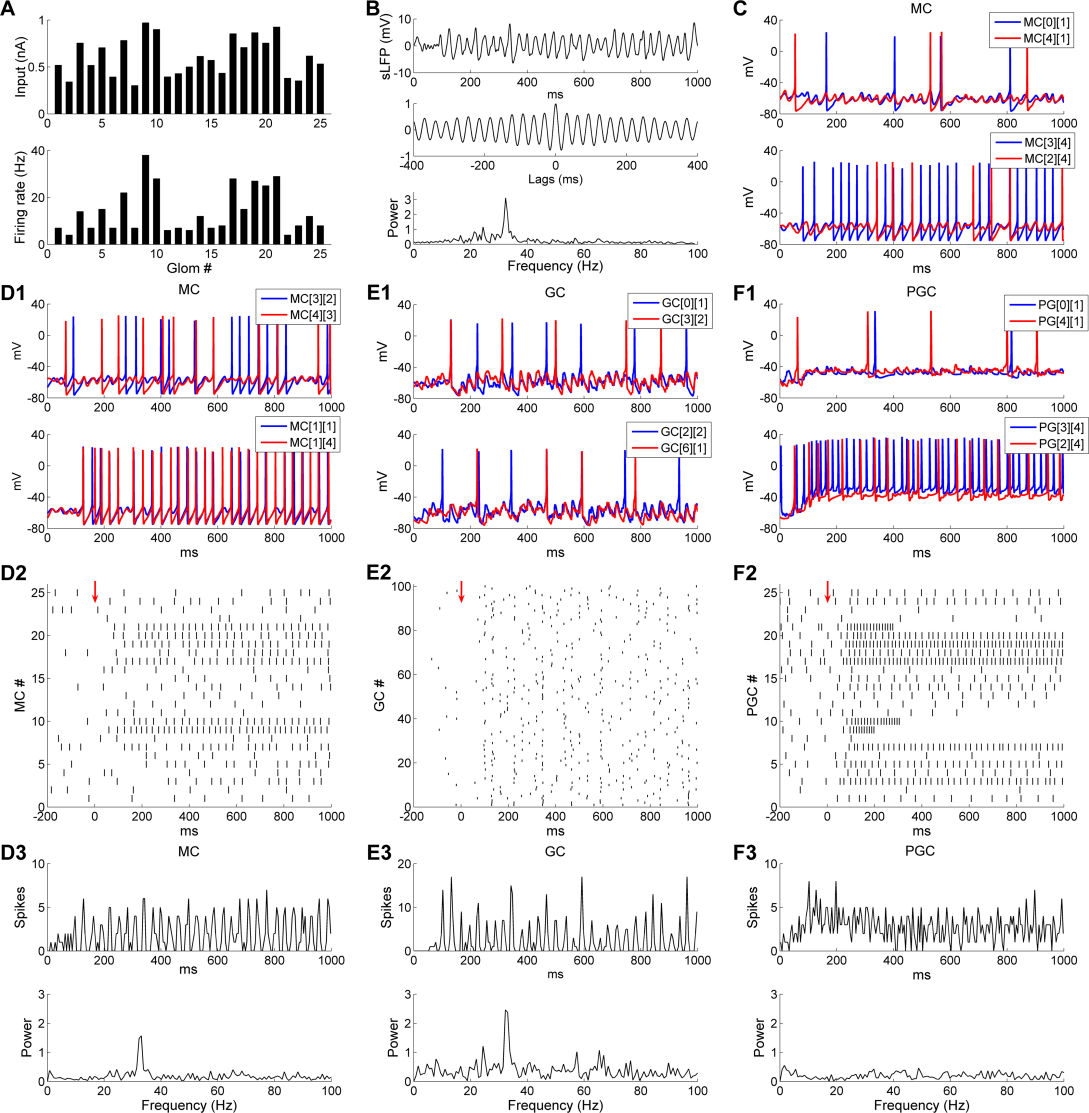
Odor stimulation induces gamma oscillation in the 2D OB model. ***A*:** Steady-state OSN input intensities (*top*) and odor-evoked firing rates (*bottom*) of all 25 mitral cells. ***B*:** Simulated LFP (*top*) during odor presentation, with autocorrelation (*middle*) and frequency power spectrum (*bottom*). ***C*:** Voltage responses during odor presentation of a pair of MCs exhibiting STOs along with sparse spikes (*top*) and another pair of MCs with one exhibiting dense spiking and the other sparse spiking with ongoing STOs (*bottom*). ***D*:** MC population spikes exhibit gamma synchrony. ***D1*:** Voltage responses of two MCs exhibiting mixed STOs and spikes (*top*) and another two MCs exhibiting dense spiking activity (*bottom*) during odor presentation. ***D2*:** Spike raster plot of MC population activity. The *red arrow* designates the onset of odor input. ***D3*:** MC population activity (*top*) with frequency power spectrum (*bottom*). Bin width is 5 ms and all MC spikes are summed in each bin. ***E*:** GC population spikes exhibit gamma synchrony. ***E1*:** Voltage responses of two typical pairs of GCs during odor presentation. ***E2*:** Spike raster plot of GC population activity. The *red arrow* designates the onset of odor input. ***E3*:** GC population activity (*top*) with frequency power spectrum (*bottom*). Bin width as in ***D***. ***F*:** PGC population spikes do not exhibit gamma synchrony. ***F1*:** Voltage responses of two typical pairs of PGCs during odor presentation. ***F2*:** Spike raster plot of PGC population activity. The *red arrow* designates the onset of odor input. ***F3*:** PGC population activity (*top*) with frequency power spectrum (*bottom*). Bin width as in ***D***.

Stimulation with simulated odorants induced gamma oscillations that were coherent across the entire OB network. Simulated odorants comprised heterogeneous levels of input delivered to the 25 MC/PGC pairs; each MC fired at a different mean rate corresponding to the strength of its afferent input (including feedforward inhibition from its associated PGC; Fig 2A). The mean MC firing frequency in response to odor stimulation was 14 Hz (min = 4 Hz; max = 38 Hz; standard deviation (SD) = 9.8 Hz). Despite these heterogeneous firing rates, a strong and broadly coherent oscillation emerged in the gamma band (32.4 Hz, Fig 2B), consistent with *in vitro* recordings from olfactory bulb [19, 35]. Individual MCs responded in a mixed mode, usually spiking at mean frequencies substantially below the underlying STO frequency, but with odor-evoked spikes phase-constrained to the underlying sLFP oscillation, as observed experimentally [10, 13]. Moreover, systemwide coherence was maintained; voltage timeseries depictions of different pairs of MCs confirmed that the STOs of different MCs were synchronized with one another (Fig 2C, *top panel*), MC spikes were synchronized with STOs from other MCs (Fig 2C, *bottom panel*), and MC spikes were also substantially synchronized with spikes from other MCs (Fig 2D1 and 2D2). GC subthreshold voltages also fluctuated rhythmically and were well synchronized with one another (Fig 2E1), as were GC spikes (Fig 2E2), despite GCs’ low mean firing rates (4.6 Hz). In contrast, no gamma-band synchrony was observed in the subthreshold voltage fluctuations (Fig 2F1) or spiking activity of PGCs (Fig 2F2). Population spike histograms of MCs, GCs and PGCs with corresponding frequency power spectra are shown in Fig 2D3, 2E3 and 2F3 respectively. The population spiking activities of both MCs and GCs exhibited gamma rhythmicity, and the frequency was the same as that measured from the sLFP (32.4 Hz; Fig 2D3 and 2E3). By comparison, no rhythmicity was observed in the PGC population spike histogram, and the frequency power spectrum was flat (Fig 2F3).

**Fig 3:**
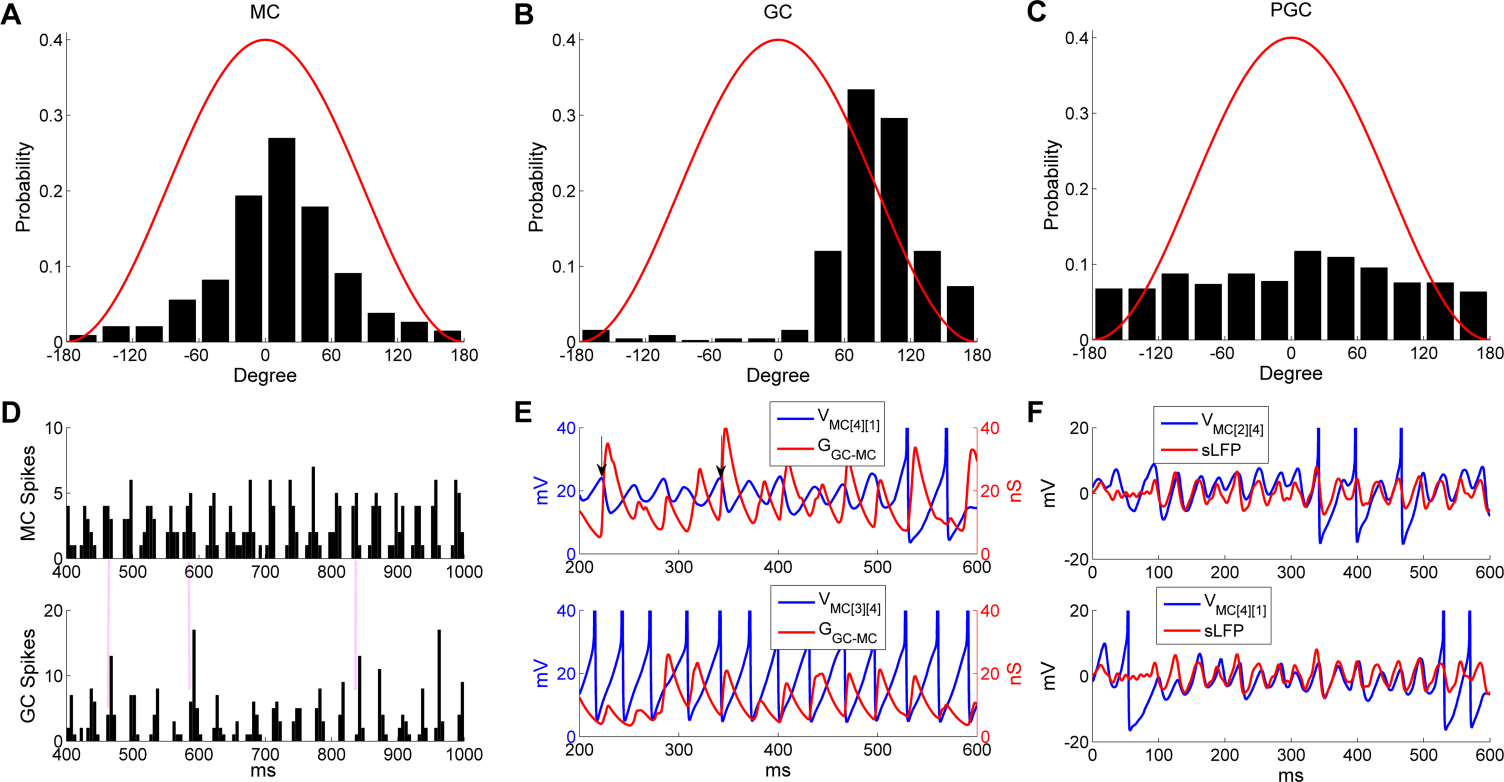
MC and GC spikes, but not PGC spikes, are phase-constrained within common gamma cycles. ***A*:** Distribution of MC spike phases with respect to sLFP oscillations. ***B*:** Distribution of GC spike phases with respect to sLFP oscillations. ***C*:** Distribution of PGC spike phases with respect to sLFP oscillations. ***D*:** Spike timing histograms of MCs (*upper*) and GCs (*bottom*). Bin width is 5 ms and all MC/GC spikes are summed in each bin. Vertical lines accentuate the alignment of spike time distributions. ***E*:** MC STOs with associated cumulative GC-mediated GABA_A_ synaptic conductance (*top*) and MC spikes with associated cumulative GC-mediated GABA_A_ synaptic conductance (*bottom*), during an odor presentation. The *vertical black arrows* indicate STO phase resets generated by GABAergic input. ***F*:** Synchronization of MC STOs with the sLFP. The MC voltage was raised by 80 mV in ***E*** and 60 mV in ***F*** for display purposes.

Examined in aggregate, MC spikes were phase-constrained within the common, coherent gamma cycle of the OB network. The majority of MC spikes were evoked near the crest of the oscillatory sLFP (Fig 3A). GC spikes also were phase-constrained within the gamma cycle, occurring predominantly during the descending phase of the sLFP (Fig 3B). In contrast, PGC spikes were not phase-constrained, but were distributed uniformly across the gamma oscillation cycle (Fig 3C).

Because of the tight phase-locking between MC/GC spikes and the sLFP cycle, the gamma rhythm also was evident in MC/GC population spiking activities (Fig 3D). The tightly alternating relationship between MC and GC population spiking, but not PGC spiking, suggested that this temporal delimiting of MC activity arose from effective feedback inhibition delivered by granule cells. To illustrate this point, we plotted the voltage traces of a weakly-activated MC and a strongly-activated MC against their respective cumulative GC-mediated GABA_A_ conductances (Fig 3E). The weakly-activated MC STO depolarized directly as its inhibitory conductance decayed (Fig 3E, *upper panel*) and the strongly-activated MC fired only after release from inhibition (Fig 3E, *lower panel*). Moreover, the inhibitory conductance increased again directly following the evocation of MC spikes, initiating the next excitation-inhibition cycle.

Sufficiently strong inhibitory GC input also effectively reset the phase of MC STOs (Fig 3E, *upper panel*, *arrows*), consistent with experimental observation and earlier cellular models [28, 37–39]. In principle, such resets erase the history imposed by longer-timescale internal dynamics, thereby enabling afferent input levels across the MC population to determine the depolarization rates in each MC from a common starting state, potentially governing MC spike phase as well as spike probability [39, 50]. Moreover, recurrent resets also serve to supersede the intrinsic frequency of MC STOs, enabling the network to oscillate at a frequency faster than that generated by intrinsic STO dynamics [35]. In aggregate, these reciprocal interactions between MCs and GCs synchronized MC internal dynamics and MC spikes, incorporating them into a coherent gamma oscillation in which MC spikes were reliably phase-constrained with respect to the common oscillatory sLFP of the network (Fig 3F; [10, 13]).

**Fig 4:**
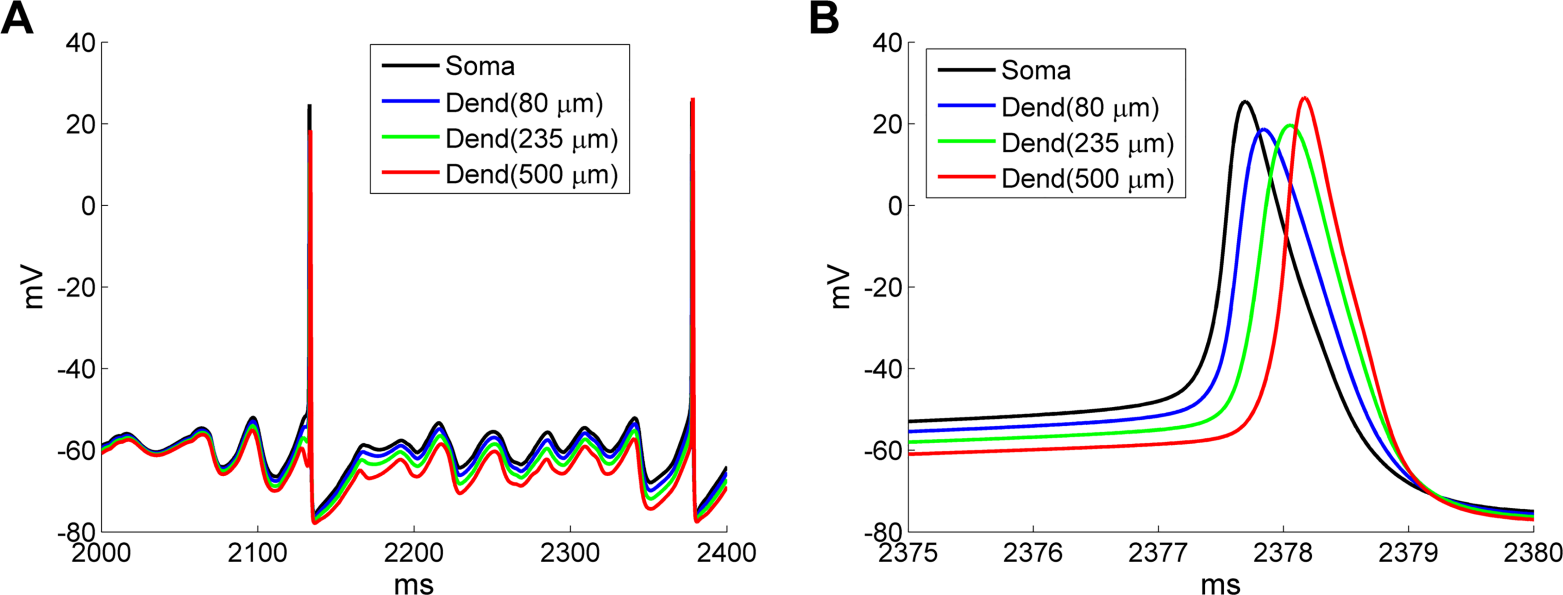
Propagation delay of MC action potentials along the lateral dendrite. ***A*:** MC membrane voltages recorded at the soma and three different locations on the lateral dendrite (80 μm, 235 μm and 500 μm from the soma). ***B*:** Expanded view of the spike propagation delay along the lateral dendrite.

Functional computations in the olfactory bulb are generally independent of the physical distance between columns [2, 51–54], though their underlying biophysical mechanisms often have proximity-dependent properties. We therefore asked whether the distance-dependent spike propagation delays along MC lateral dendrites were sufficiently heterogeneous to impair the global coherence of gamma oscillations across the OB circuit. To visualize the propagation delay as a function of distance, the membrane potentials of a representative MC (MC[2][2]) were recorded from the soma and from the locations of three reciprocal synapses distributed along the lateral dendrite (at 80 μm, 235 μm, and 500 μm from the soma; Fig 4). These three synapses connected, respectively, to an adjacent GC (GC[5][4]), a GC connecting near the middle of the lateral dendrite (GC[6][3]), and a GC connecting at the end of the lateral dendrite (GC[0][1]). Subthreshold activity in the MC dendrite was slightly hyperpolarized as the recording site progressed away from the soma, but spikes propagated at essentially full amplitude (Fig 4A). Spike propagation was rapid, with less than 1 ms delay from the soma to the end of the 500 μm dendrite (Fig 4B), suggesting that heterogeneous spike propagation delays would have little effect on network synchronization. This reflects the experimental observation that spikes fully propagate along a MC lateral dendrite with little delay (Fig 2 in [55]), and is consistent with previous computational work in which spike backpropagation along MC lateral dendrites activates granule cells independently of distance [56].

### Mitral cell STOs enhance network synchronization

**Fig 5:**
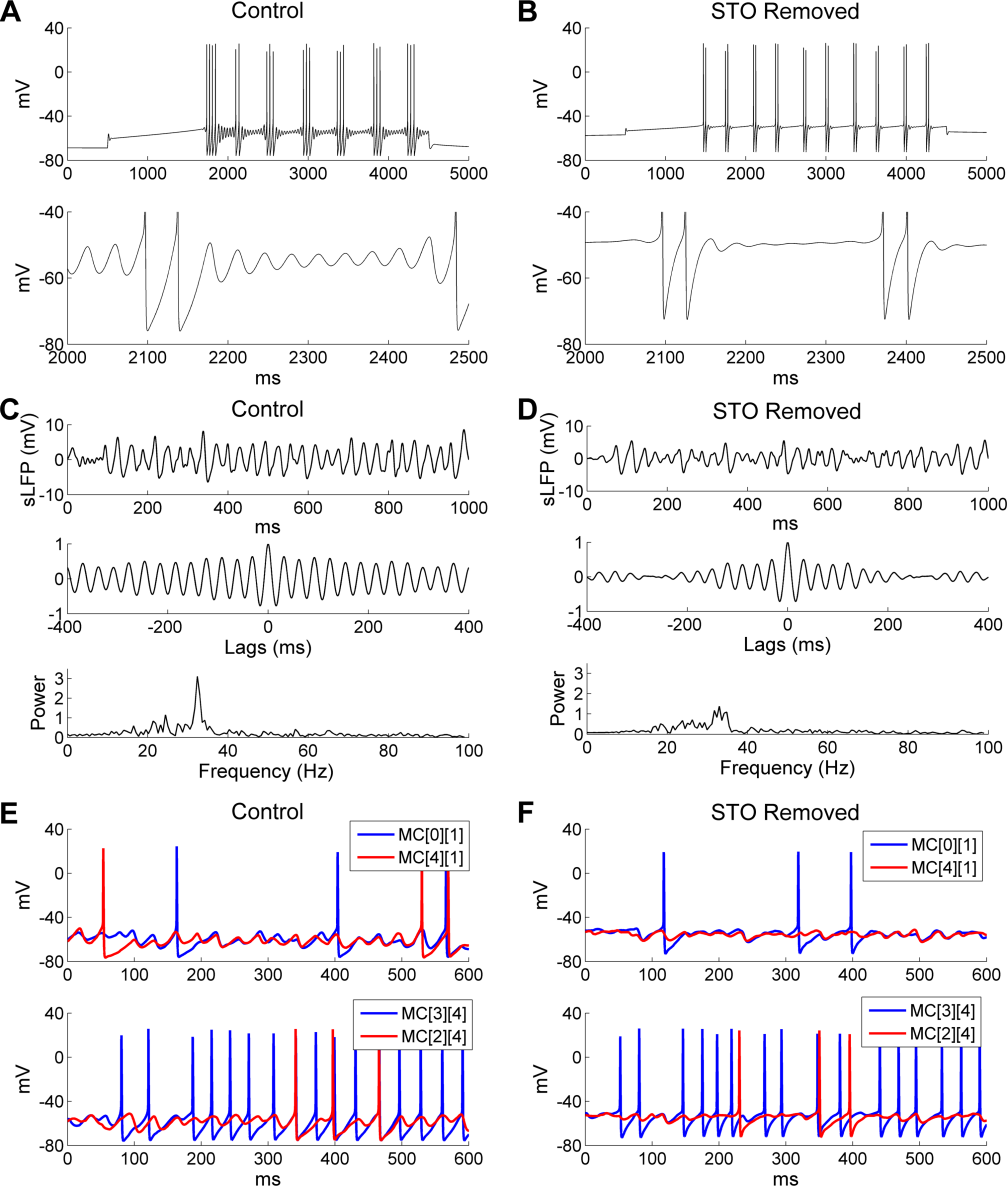
Removing MC STOs impairs OB gamma oscillations. ***A*:** Voltage response of an isolated MC model cell to a 0.2 nA current injection (*top*) and an expanded view of the STOs (*bottom*) under control conditions. ***B*:** As in ***A***, but after MC STOs were removed. ***C*:** Simulated LFP (*top*) during odor presentation, with autocorrelation (*middle*) and frequency power spectrum (*bottom*) under control conditions (same as **Fig 2B**). ***D*:** As in ***C*,** but after MC STOs were removed. ***E*:** Voltage responses of two pairs of MCs under control conditions. ***F*:** As in ***E***, but after MC STOs were removed. The MC STOs were removed by replacing the persistent sodium current (*I*_NaP_) with an ohmic cation current; the conductance of this current was tuned to maintain the same firing frequency.

It has been proposed that gamma oscillations in the OB depend on MC STOs [28, 33]; however, in the PING framework, the pyramidal (excitatory) neurons generally do not exhibit resonance. We therefore asked whether and how MC STOs contribute to the robustness, power, and regularity of gamma coherence and spike synchronization in the active OB network. To investigate this, we first removed STOs from model MCs and examined the effect of this change on network dynamics. Specifically, STOs were eliminated by replacing the persistent sodium current (*I*_NaP_) in all MCs of the network with ohmic cation currents scaled to maintain the same MC firing rates under the same current injection levels (Fig 5A and 5B; [39]). The cation current was modeled as *I*_*CAT*_ = *g*_*CAT*_(*v*−*E*_*CAT*_), where *g*_*CAT*_ = 0.26 *mS*/*cm*^2^ and *E*_*CAT*_ = 0 *mV*. Under this manipulation, the power and regularity of odor stimulus-induced network gamma were substantially reduced and sLFP oscillations became less coherent, as evidenced by reduced persistence in the autocorrelogram and a lower, flatter peak in the power spectrum (compare Fig 5C with Fig 5D). An examination of membrane potential timeseries from two pairs of MCs revealed that, although MCs without intrinsic STOs could still display subthreshold voltage fluctuations owing to phasic inhibition from granule cells, these fluctuations had smaller amplitudes and were much less regular compared with intact STOs in control cells (compare Fig 5E with Fig 5F). MC spikes also became less synchronized with one another in the absence of intrinsic STOs (compare Fig 5E with Fig 5F, *bottom panels*), although the mean odor-evoked MC firing rates were essentially identical (*Control*: 14 Hz; *STO removed*: 13.2 Hz). Finally, the synchronization index (*SI*) was reduced from 0.64 in controls to 0.53 when STOs were removed. Hence, MC resonance contributed substantially to the integrity and regularity of coherent gamma oscillations in the active OB network, even when the intrinsic STO frequency was superseded by the PING-like mechanisms of the network frequency (see below). The added stability and robustness of OB gamma oscillations contributed by these MC resonance properties resembles the advantages of resonance-induced gamma (RING) oscillations [57], with the important distinction that RING is described for resonant inhibitory interneurons, whereas in the OB network it is the excitatory principal neurons that are resonant. The mechanism of OB oscillations can be described as pyramidal resonance interneuron network gamma (PRING), thereby acknowledging the PING-like properties of the activated gamma oscillation as well as the additional properties afforded by MC resonance.

### Faster network time constants supersede intrinsic STO frequencies during sensory activation

**Fig 6:**
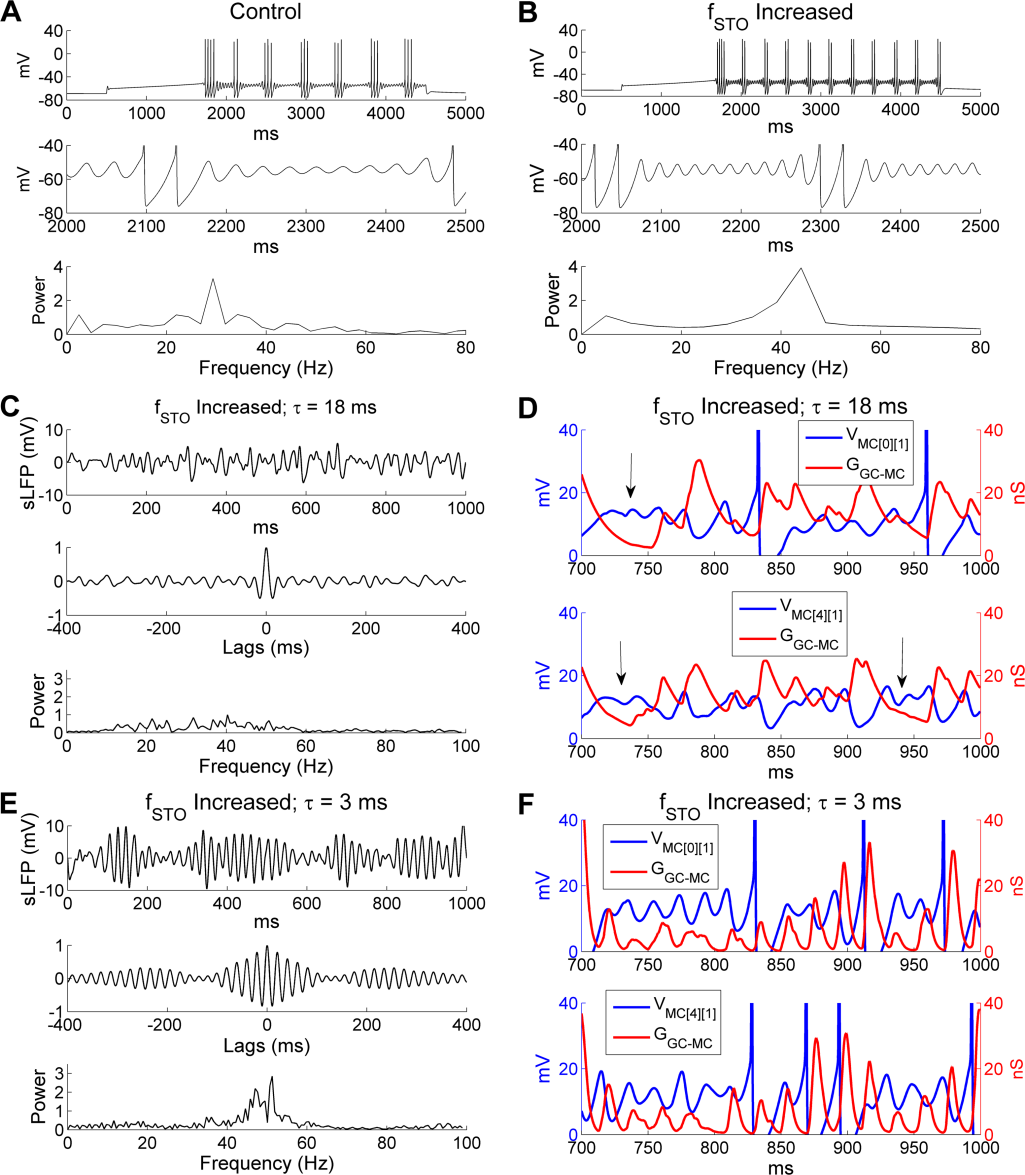
Higher intrinsic MC STO frequencies require faster GABAergic synaptic decay to synchronize activity. ***A*:** Voltage response of an isolated MC model cell to a 0.2 nA current injection (*top*), an expanded view of the STOs (*middle*), and the STO frequency power spectrum (*bottom*) under control conditions. ***B*:** As in ***A***, but with a higher intrinsic STO frequency. ***C*:** Simulated LFP (*top*) during odor presentation, with autocorrelation (*middle*) and frequency power spectrum (*bottom*) under conditions in which the STO frequency was increased while the decay time constant of the GC-mediated GABA_A_ synaptic conductance remained unchanged (18 ms). ***D*:** Plot of MC STOs with associated cumulative GC-mediated GABA_A_ synaptic conductance when STO frequency was increased. The vertical black arrows indicate multiple STO cycles during one single GABA_A_ conductance decay. ***E*:** As in ***C***, but when the decay time constant of the GC-mediated GABA_A_ conductance was reduced to 3 ms (from 18 ms) in the presence of the higher intrinsic STO frequency. ***F*:** As in ***D***, but when the decay time constant of the GC-mediated GABA_A_ conductance was reduced to 3 ms in the presence of the higher intrinsic STO frequency. The MC STO frequency was increased by reducing the activation time constant of the slow-inactivating potassium current (*I*_KS_) while increasing the maximal conductances of *I*_NaP_ and *I*_KS_ to maintain the same firing frequency. The MC voltage was raised by 70 mV in ***D*** and ***F*** for display purposes.

Intrinsic STO frequencies appear to yield to higher-frequency network-based oscillations owing to recurrent STO phase resets delivered by GC-mediated synaptic inhibition [28, 35]. If this interpretation is correct, and MC resonance is an important contributor to network coherence and frequency stability, then this oscillatory coherence should be disrupted if the intrinsic STO frequency becomes faster than the natural frequency of the synaptically-based network oscillation. To test this, we increased the intrinsic MC STO frequency by reducing the time constant of the activation variable of the slow potassium current (*I*_KS_) [33]. Specifically, we reduced the activation time constant of *I*_KS_ from 10 ms to 5 ms, and increased the conductance densities of the *I*_KS_ and *I*_NaP_ currents by factors of 1.6 and 1.3 respectively to maintain approximately the same STO amplitudes and MC firing rates. These modifications increased the STO frequency in an isolated MC model cell from 29 Hz (in controls) to 44 Hz in response to a 200 pA depolarizing current injection (Fig 6A and 6B). Without altering any other model parameters, this change in the intrinsic STO frequency seriously disrupted the sLFP gamma rhythm and sharply reduced gamma power in the OB network (compare Fig 6C with Fig 5C). A comparison of STO voltage timeseries with the aggregated GABA_A_ conductances in the same MCs confirmed that GC inhibition could no longer effectively regulate MC STOs, which became irregular (Fig 6D). Phase locking between MC spikes and sLFP oscillations also was significantly reduced (*SI*, controls: 0.64; *increased STO frequency*: 0.38), although the average odor-evoked MC firing rate was virtually unchanged (controls: 14 Hz; *increased STO frequency*: 14.4 Hz).

If this disruption was due to a mismatch between intrinsic STO frequency and the natural frequency of the network oscillation, as predicted, rather than to some separate effect of the changes made to the model MCs, then the coherence of OB gamma oscillations should be restored if the natural frequency of the network oscillation was also increased so as to again be faster than those of the MC STOs. In PING and ING networks, the natural frequency of network oscillations depends strongly on the decay time constant of the inhibitory synapse [32, 58]. Indeed, when the GABA_A_ receptor decay time constant of the GC→MC synapses was reduced to 3 ms (from the default 18 ms), in a network populated with MCs exhibiting the higher intrinsic STO frequency, a strong gamma oscillation re-emerged at 51.3 Hz (Fig 6E)–considerably faster than the 32.4 Hz frequency exhibited by control networks. Under these conditions, MC STOs again displayed rhythmicity and were entrained effectively by GC-mediated GABA_A_ synaptic conductances (Fig 6F); network synchrony also was substantially restored (*SI*, controls: 0.64; increased STO frequency alone: 0.38; increased STO frequency + 3 ms synaptic decay time constant: 0.56). These simulations indicate that the decay rate of GC-mediated GABA_A_ inhibition must be faster than the intrinsic MC STO frequency in order to be able to synchronize MC dynamics.

**Fig 7:**
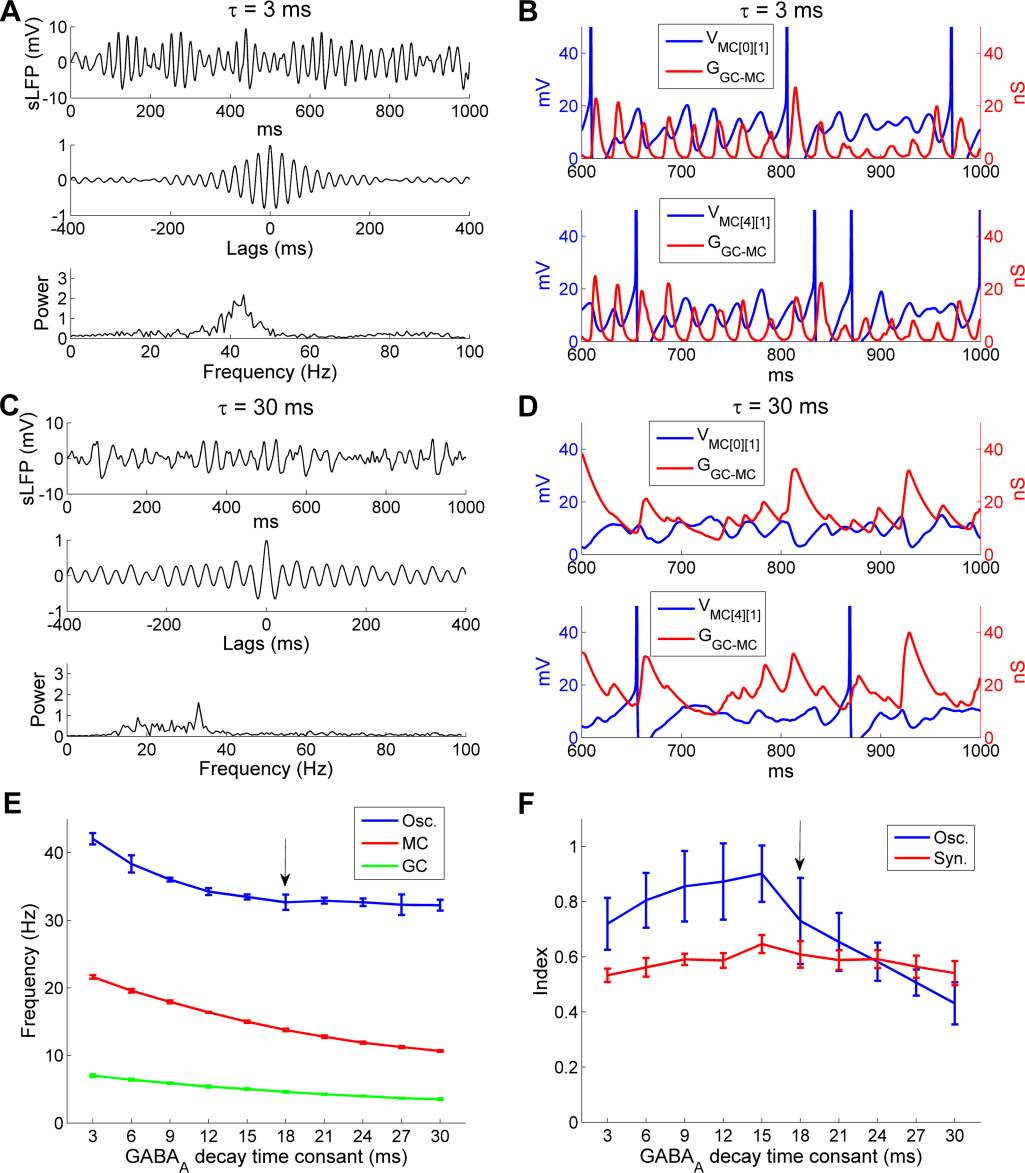
The OB gamma oscillation frequency is responsive to faster, but not slower, GABA_A_ decay time constants. ***A*:** Simulated LFP (*top*) during odor presentation, with autocorrelation (*middle*) and frequency power spectrum (*bottom*), when the decay time constant of the GC-mediated GABA_A_ conductance was reduced from 18 ms to 3 ms. ***B*:** Plot of MC STOs with associated cumulative GC-mediated GABA_A_ synaptic conductance when the latter had a decay time constant of 3 ms. ***C*:** As in ***A***, but when the decay time constant of the GC-mediated GABA_A_ conductance was increased from 18 ms to 30 ms. ***D*:** As in ***B***, but when the decay time constant of the GC-mediated GABA_A_ conductance was increased from 18 ms to 30 ms. ***E*:** Average odor-evoked MC and GC firing rates and sLFP oscillation frequency as functions of the decay time constant of the GC-medicated GABA_A_ conductance. ***F*:** Synchronization and oscillation indices as functions of the decay time constant of the GC-medicated GABA_A_ conductance. The default (control) decay time constant in this study was 18 ms (indicated by black arrows in ***E***, ***F***). Error bars denote standard deviations (SD). The MC voltage was raised by 70 mV in ***B*** and ***D*** for display purposes.

To test whether it was important that the inhibitory synaptic decay time constant be closely matched to the MC STO frequency, or that it simply be faster, we tested a network in which we paired the faster (3 ms) GABA_A_ synaptic decay time constant with the default (29 Hz) intrinsic MC STO frequency. Under these parameters, the network oscillation frequency increased from 32.4 Hz (under control conditions; Fig 2B) to 43.4 Hz (Fig 7A). This increase in oscillation frequency was accompanied by a slight reduction in oscillatory power and coherence, as indicated by a wider spectral peak and less persistent periodicity, though the amplitudes of the two spectral peaks were comparable (compare Fig 7A with Fig 2B). Additionally, under these conditions the GABA_A_ conductance fluctuated regularly and decayed fully within every gamma cycle owing to its fast dynamics, effectively entraining MC STOs (Fig 7B). In contrast, when the GABA_A_ decay time constant was increased from 18 ms to 30 ms, there was no change in the peak frequency of the network oscillation (18 ms: 32.4 Hz; 30 ms: 33 Hz), though its power was reduced considerably (compare Fig 7C with Fig 2B). Within individual MCs, the slowly decaying GABA_A_ conductance accumulated across successive gamma cycles and lost much of its rhythmicity, resulting in inconsistent effects on MCs that failed to supersede their intrinsic STO frequency preferences (Fig 7D). Accordingly, under control parameters, synaptic decay time constants faster than ~18 ms progressively increased network sLFP oscillation frequencies, whereas slower time constants had no effect (Fig 7E). These faster kinetics also maintained relatively high power spectral peaks at the gamma frequency (oscillation indices), whereas slower synaptic kinetics resulted in rapidly declining oscillation indices (Fig 7F). Mean spiking frequencies in both MCs and GCs, however, varied monotonically with respect to the rate of GABA_A_ decay (Fig 7E), likely because slower decay rates produced an overall increase in the total integrated inhibition of MCs. Specifically, as the decay time constant was increased from 3 ms to 30 ms, the MC firing rate decreased from 21.6 Hz to 10.8 Hz, resulting in a concomitant decrease in GC firing rate from 6.9 Hz to 3.4 Hz. In sum, these results showed that a wide range of synaptic decay time constants generated reliable coherence from the OB network provided that they were lower (faster) than a threshold value determined by the intrinsic frequency of MC STOs. However, there also was a clear peak (15 ms; Fig 7F), indicating that the strongest network oscillatory power could be achieved by an optimal matching of the synaptic and STO timescales. Additionally, these results demonstrated that the network oscillation frequency was robust to substantial changes in mean spike frequencies in both MCs and GCs.

**Fig 8:**
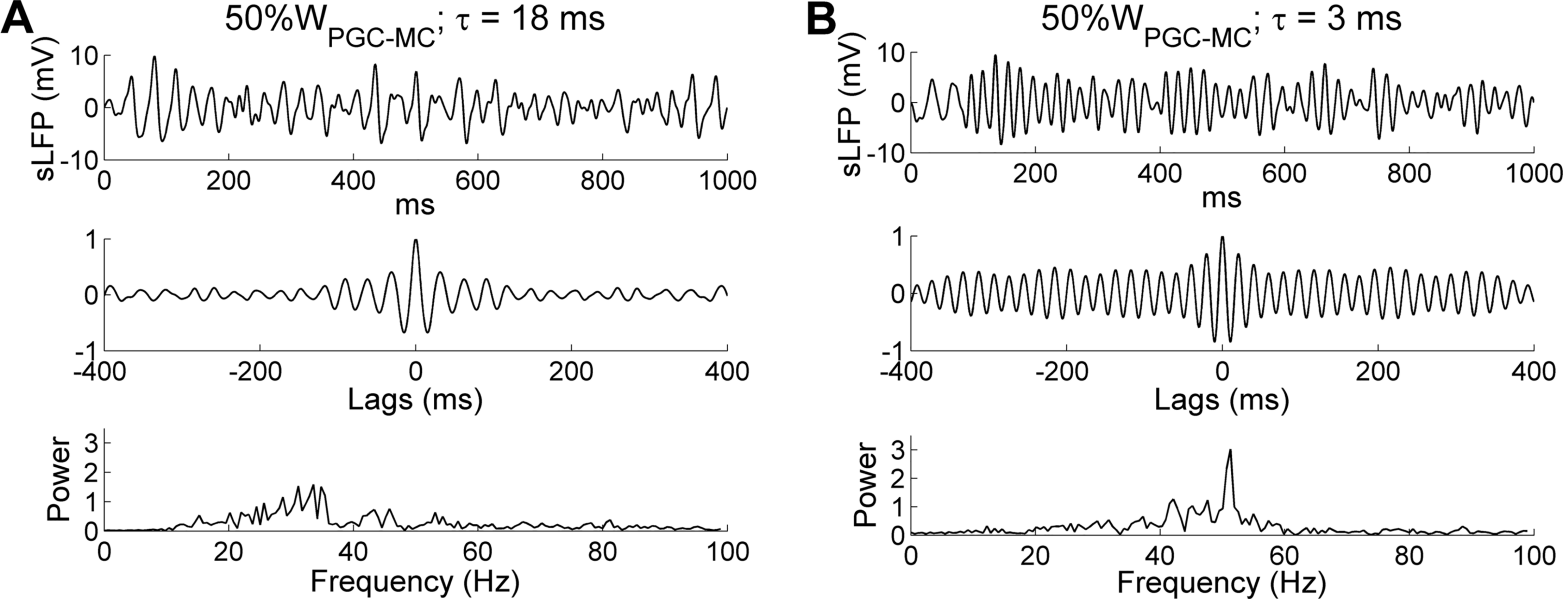
Gamma oscillation frequency is determined by the decay time constant of the GC-mediated GABA_A_ synaptic conductance. ***A*:** Simulated LFP (*top*) during odor presentation, with autocorrelation (*middle*) and frequency power spectrum (*bottom*), when PGC→MC synaptic weights were reduced by 50% while maintaining the same decay time constant (18 ms). ***B*:** As ***A*,** but with a reduced decay time constant of the GC-mediated GABA_A_ conductance (3 ms) in addition to a 50% reduction in PGC→MC synaptic weights.

Finally, we decided to increase the intrinsic MC STO frequency by increasing the excitation levels of all MCs, rather than by altering their *I*_KS_ and *I*_NaP_ conductance parameters as above, in order to test whether similar dynamical effects resulted. Depolarizing MCs increases their intrinsic STO frequencies both experimentally [28] and in the present model. To broadly increase MC excitation while retaining the same heterogeneous odor inputs, we decreased the level of PGC-mediated inhibition on MCs by reducing the PGC→MC synaptic weight to half of the control value (from 4 to 2). The results largely conformed to those observed when STO frequencies were increased by adjusting cellular conductance parameters (Fig 6). Under default parameters (with an 18 ms GABA_A_ decay time constant), reducing PGC inhibitory weights by half had no effect on the network oscillation frequency (controls: 32.4 Hz; 50%W_PGC-MC_: 33.6 Hz), but did impair the coherence and stability of field potential oscillations and reduce the oscillation index (peak spectral power; compare Fig 8A with Fig 2B). In contrast, when using a faster GC synaptic decay time constant of 3 ms, this reduced PGC inhibition produced a coherent gamma oscillation at a higher peak frequency (controls: 32.4 Hz; 3 ms decay time constant only: 43.4 Hz; 3 ms decay time constant + 50%*W*_PGC-MC_: 51.3 Hz; Fig 8B), because the faster synaptic decay was again able to effectively reset the intrinsic MC STOs on every cycle. This result further suggests that higher overall levels of MC excitation, which generate faster intrinsic STO dynamics, would require correspondingly faster synaptic inhibition kinetics in order to maintain network stability, and thereby demonstrates the importance of maintaining a limited range of mean MC activity levels via global afferent activity normalization ([59]; corrected mechanism in [54]).

### Optimal inhibitory synaptic weights are required for strong and coherent OB gamma oscillations

**Fig 9:**
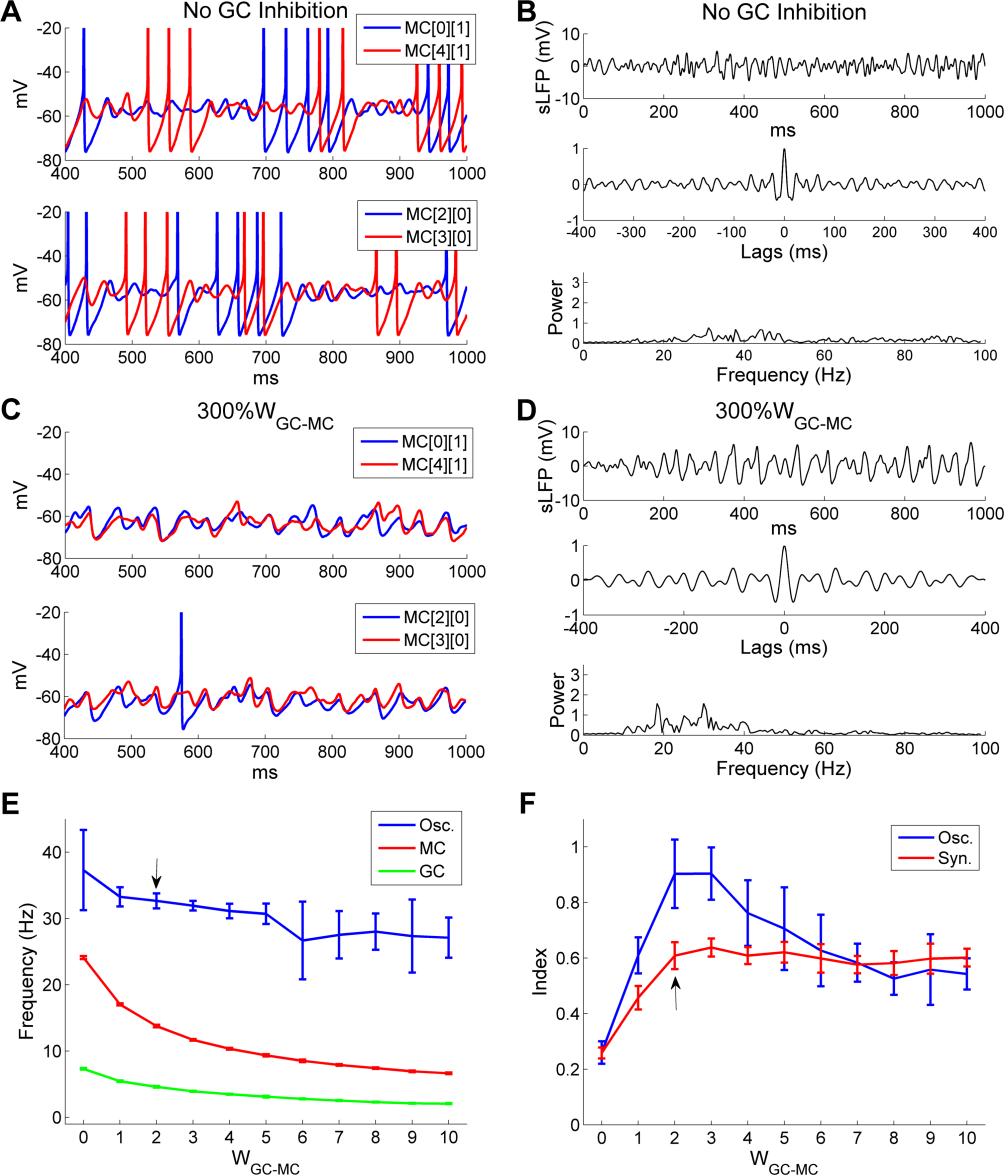
There is an optimal GC→MC synaptic strength for strong and coherent OB gamma oscillation. ***A*:** Membrane potential timeseries of two pairs of MCs during odor presentation without GC inhibition. ***B*:** Simulated LFP (*top*) during odor presentation, with autocorrelation (*middle*) and frequency power spectrum (*bottom*), in the absence of GC inhibition. ***C*:** As in ***A***, but with a 3-fold increase of the GC→MC synaptic weight (300%*W*_GC-MC_). ***D*:** As in ***B***, but with 300%*W*_GC-MC_. ***E*:** Average odor-evoked MC and GC firing rates and sLFP oscillation frequency as functions of GC→MC synaptic weights. ***F*:** Synchronization and oscillation indices as functions of GC→MC synaptic weights. The default GC→MC synaptic weight in this study was 2 (indicated by black arrows in ***E***, ***F***). Error bars denote standard deviations (SD).

The synaptic weight of GC→MC inhibition also is an important factor in determining the stability of network gamma oscillations. To assess this effect, we varied the GC→MC synaptic weight (*W*_*GC*_→_*MC*_) from zero (full blockade) up to five times the default value. Under full blockade conditions, MC spikes and STOs were desynchronized (Fig 9A) and network sLFP oscillations were dramatically reduced (compare Fig 9B with Fig 2B). Whereas overall spike rates increased substantially (average odor-evoked spike rate, controls: 14 Hz; no GC inhibition: 24 Hz), synchronization among MC spikes was sharply reduced (*SI*, controls: 0.63; no GC inhibition: 0.30). These results further confirm that GC-mediated feedback inhibition is necessary for the synchronization of mitral cells into a coherent gamma rhythm in the OB.

In contrast, when *W*_*GC*_→_*MC*_ was increased threefold (from 2 to 6), MC spiking activity was reduced substantially (controls: 14.0 Hz; 300% *W*_*GC*_→_*MC*_: 8.4 Hz) and STOs were corrupted by an irregular mixture of shorter and longer oscillation periods, though MC membrane potential fluctuations were still moderately well-coordinated (Fig 9C). The frequency power spectrum reflected this disruption, presenting a number of low-power peaks (Fig 9D); two of these (at 18.3 Hz and 29.9 Hz) were somewhat more distinct, though both remained well below control amplitudes (compare Fig 9D with Fig 2B). These results indicate that excessive inhibition of MCs by large GC→MC synaptic weights impairs network gamma oscillations by disrupting STO periodicity.

The frequency of the network sLFP oscillation and the mean spike rates of both MCs and GCs declined as *W*_*GC*_→_*MC*_ increased from 0 to 6 and remained stable thereafter (Fig 9E). In contrast, the synchronization index rose substantially as *W*_*GC*_→_*MC*_ increased from 0 to 2 and maintained this level for all larger synaptic weights measured (Fig 9F). The oscillation index (spectral peak amplitude) also increased greatly as *W*_*GC*_→_*MC*_ grew from 0 to 2, but then progressively decreased once *W*_*GC*_→_*MC*_ exceeded 3 (Fig 9F). This pattern of results indicates that the degradation of gamma oscillatory power at larger GC→MC weights was not a result of reduced phase coupling, but of disrupted STO periodicity (Fig 9C). In sum, while sufficient GC inhibition is required to reset and synchronize MC STOs, excessive GC synaptic weights are detrimental to the stability of the gamma rhythm; an optimal level of GC inhibition is required to sustain a strong and coherent gamma oscillation.

**Fig 10:**
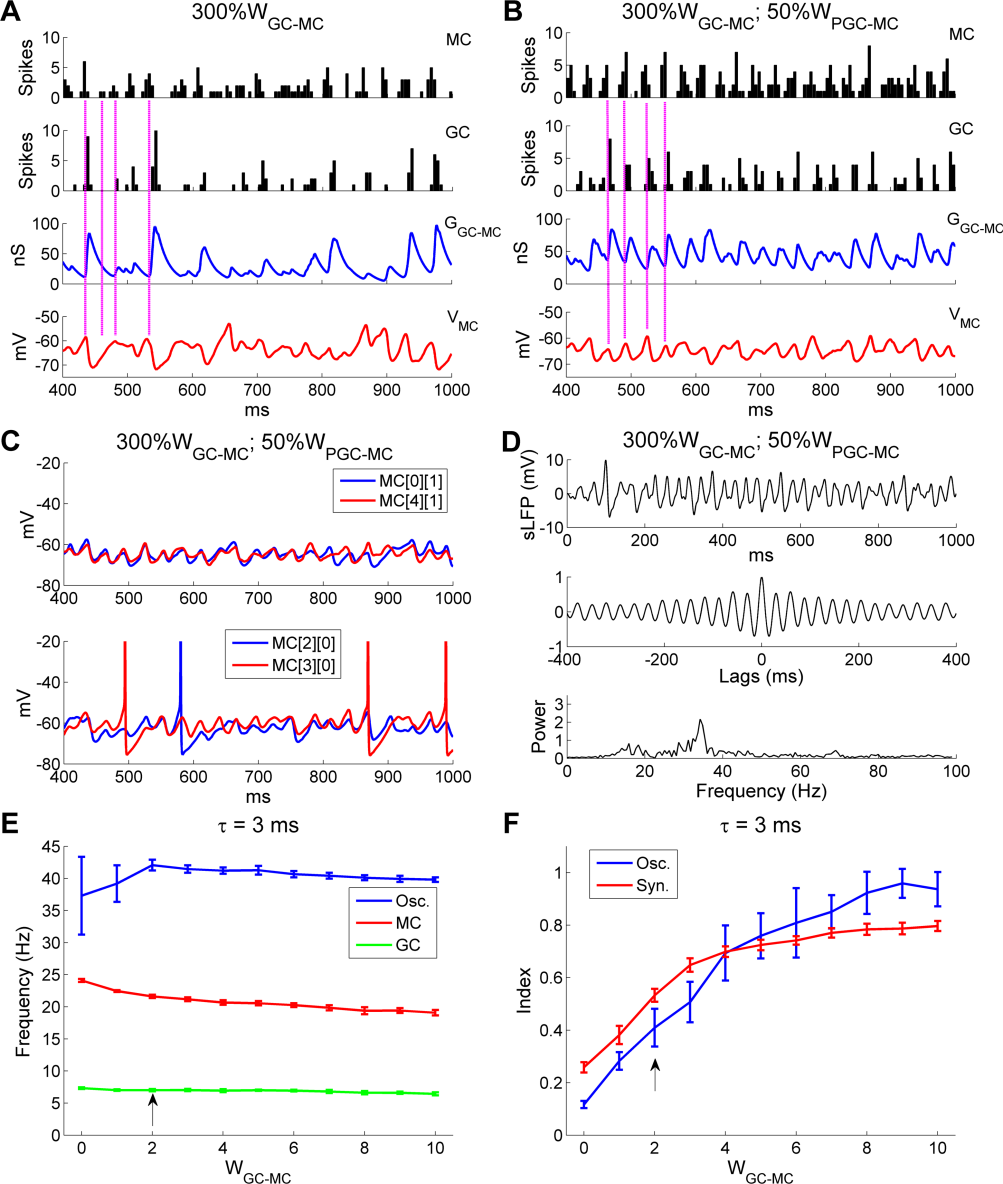
OB gamma oscillation impairments arising from excessive GC→MC synaptic weights can be counteracted by reducing PGC inhibition of MCs. ***A*:** Spike timing histograms of MCs (*top panel*) and GCs (*second panel*) with associated cumulative GC-mediated GABA_A_ synaptic conductance (*third panel*) and MC STOs (*bottom panel*) when the GC→MC synaptic weight was increased threefold (300%*W*_GC-MC_). ***B*:** As in ***A***, but with an additional 50% reduction in the PGC→MC synaptic weight (50%*W*_PGC-MC_). ***C*:** Membrane potential timeseries of two pairs of MCs during odor presentation with 300%*W*_GC-MC_ and 50%*W*_PGC-MC_. ***D*:** Simulated LFP (*top*) during odor presentation, with autocorrelation (*middle*) and frequency power spectrum (*bottom*), with 300%*W*_GC-MC_ and 50%*W*_PGC-MC_. ***E*:** Average odor-evoked MC and GC firing rates and sLFP oscillation frequency as functions of GC→MC synaptic weight when the decay time constant of the GC-mediated GABA_A_ conductance was reduced to 3 ms (from 18 ms). ***F*:** Synchronization and oscillation indices as functions of GC→MC synaptic weight when the decay time constant of the GC-mediated GABA_A_ conductance was reduced to 3 ms (from 18 ms). The default GC→MC synaptic weight in this study was 2 (indicated by *black arrows* in ***E***, ***F***). Error bars denote standard deviations (SD).

To understand in detail why larger GC→MC synaptic weights impaired gamma rhythmicity, we plotted spike time histograms for both MCs and GCs alongside the membrane potential timeseries of a representative MC and the aggregate GC-mediated GABA_A_ conductance of that MC, all under the disruptive conditions of a 3-fold increase in *W*_*GC*_→_*MC*_ (Fig 10A; same parameters as Fig 9C and 9D). At a timepoint marking a surge of synchronous MC spiking activity, GCs responded in turn with higher-than-average activity (Fig 10A, *top and second panels*, *leftmost vertical line*). Because of the large *W*_*GC*_→_*MC*_, this surge in GC activity evoked a particularly enlarged (and correspondingly persistent) GABAergic chloride conductance in MCs (Fig 10A, *third panel*, *leftmost vertical line*), which substantially hyperpolarized MC membrane potentials (Fig 10A, *bottom panel*, *leftmost vertical line*) and, in aggregate, noticeably suppressed MC firing across the network (Fig 10A, *top panel*, *second vertical line*). This reduced level of MC activity, in turn, did not induce any GC spiking in that cycle (Fig 10A, *second panel*, *second vertical line*). As the GABAergic chloride conductance continued to decay (Fig 10A, *third panel*, *second vertical line*), marginally increased numbers of spikes were generated from the MC population (Fig 10A, *top panel*, *third vertical line*), which evoked weak responses in GCs (Fig 10A, *second panel*, *third vertical line*) and hence much smaller GABAergic conductances that only minimally hyperpolarized MC membrane potentials (Fig 10A, *third and bottom panels*, *third vertical line*). After a few such “small” cycles, the MCs recovered from the effects of accumulated inhibition and a high-activity cycle occurred again (Fig 10A, *all panels*, *rightmost vertical line*). The irregularity of this recurrent process substantially distorted MC subthreshold activity and gamma rhythmicity (Fig 9D; Fig 10A, *bottom panel*).

If this disruption of gamma oscillations indeed resulted from an oversuppression of MCs by excessive GC inhibition, as hypothesized, then boosting MC excitability should restore the rhythmicity. To test this, we increased MC mean firing rates back to the control level by reducing the PGC→MC inhibitory synaptic weight (*W*_*PGC*→*MC*_) to 50% of its default value (controls: 14.0 Hz; 300% *W*_*GC*→*MC*_: 8.4 Hz; 300%*W*_*GC*→*MC*_ + 50%*W*_*PGC*→*MC*_: 14.4 Hz). GC firing rates also were restored to control levels by this change (controls: 4.6 Hz; 300%*W*_*GC*→*MC*_: 2.8 Hz; 300%*W*_*GC*→*MC*_ + 50%*W*_*PGC*→*MC*_: 4.5 Hz). Under these restored excitability conditions, MC spikes again reliably drove substantial GC responses in every gamma cycle, the GABAergic synaptic conductance changes became more regular, and the periodicity of MC subthreshold activity was substantially improved (compare Fig 10B with Fig 10A). Moreover, MC STOs were again well synchronized, and exhibited greater stability and regularity than under conditions of elevated GC inhibition but default PGC inhibition (compare Fig 10C with Fig 9C). As a result, the second spectral peak observed in Fig 9D was eliminated and a single coherent gamma peak again appeared at 34.2 Hz, comparable to the control value of 32.4 Hz (compare Fig 10D with Fig 2B). The above simulation demonstrates that the detrimental effect of excessive GC inhibition on gamma rhythmicity can be ameliorated by reduced PGC inhibition, indicating that an overall balance of excitation and inhibition is required for coherent, stable network gamma oscillations.

Finally, the synaptic weights and decay time constants of GABA_A_ synapses are not functionally independent of one another; shorter decay time constants generate less total MC inhibition and a weaker and shorter suppressive effect, all else being equal. We therefore asked whether the optimal inhibitory synaptic weights for robust oscillations and synchronization would differ depending on the synaptic time constant. We generated a network in which the GABAergic decay time constant was reduced from 18 ms (in controls) to 3 ms (as depicted in Fig 7A and 7B), and measured network oscillation and spike frequencies and the oscillation and synchronization indices as functions of GC→MC synaptic weight. As predicted, the oscillation index (*OI*) peak and the *SI* plateau both occurred at substantially higher inhibitory synaptic weights when using the faster decay time constants (compare Fig 10E and 10F to Fig 9E and 9F). The inhibitory synaptic decay time constant therefore also must be factored into the balance between excitation and inhibition that enables stable and coherent gamma oscillations across the OB network.

### Oscillatory dynamics are tolerant of increased excitatory synaptic weights

**Fig 11:**
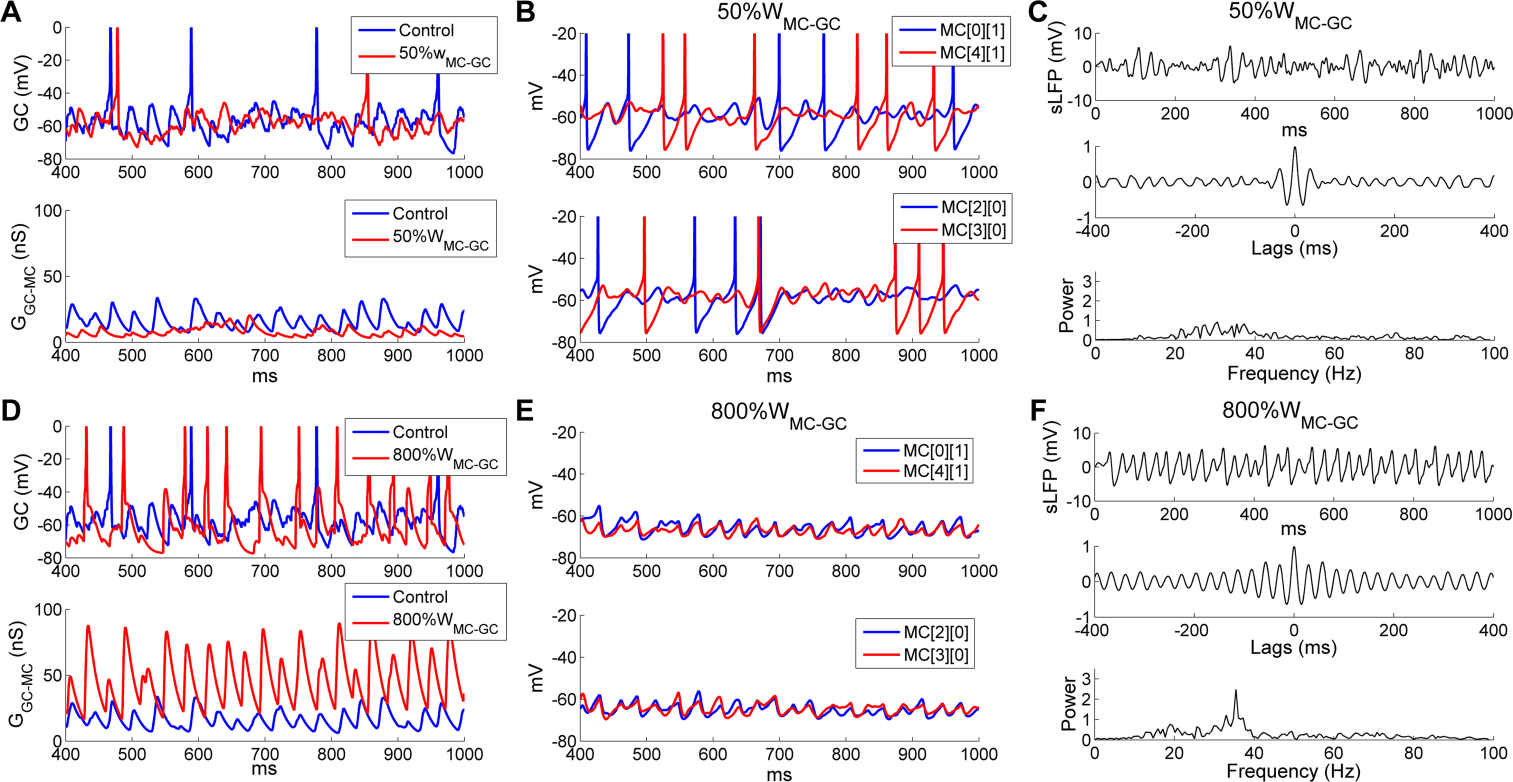
OB gamma oscillations are robust to strongly increased, but not reduced, MC→GC synaptic weights. ***A*:** Voltage timeseries of representative GCs (*top*) and GC-mediated GABA_A_ synaptic conductances on MCs (*bottom*), under control conditions and following a 50% reduction of MC→GC synaptic weights (50%*W*_MC-GC_). ***B*:** Membrane potential timeseries of two pairs of MCs with 50%*W*_MC-GC_. ***C*:** Simulated LFP (*top*) during odor presentation, with autocorrelation (*middle*) and frequency power spectrum (*bottom*), with 50%*W*_MC-GC_. ***D*:** As in ***A***, but under control conditions compared with an eightfold increase in the MC→GC synaptic weight (800%*W*_MC-GC_). ***E*:** As in ***B***, but with 800%*W*_MC-GC_. ***F*:** As in ***C***, but with 800%*W*_MC-GC_.

The functional efficacy of feedback inhibition in the OB EPL depends on the synaptic weights of both the inhibitory GC→MC and the excitatory MC→GC synapses. If a balance between excitation and inhibition is required for strong and stable gamma oscillations across the OB, then an optimal range of excitatory MC→GC synaptic weights may also exist. However, because MC→GC synapses onto adult-born GCs are plastic [47, 48], the EPL network would be expected to tolerate a substantial range and heterogeneity among these synaptic weights. To examine the functional range of synaptic weights for the excitatory MC→GC synapses in this network, we varied the MC→GC synaptic weight (*W*_*MC*→*GC*_) from 0 up to 8 times the default value. When these synapses were blocked (i.e., *W*_*MC*→*GC*_ = 0), GCs were largely inactive (0.9 Hz spontaneous background activity); other simulation results were similar to those obtained when blocking GABAergic synaptic transmission (i.e., *W*_*GC*→*MC*_ = 0; Fig 9A and 9B) and are not separately reported here. When *W*_*MC*→*GC*_ was reduced to 50% of the default value (from 1 to 0.5), the GC subthreshold potential was substantially hyperpolarized and lost much of its rhythmicity compared with controls (Fig 11A, *upper panel*), leading to significantly smaller and arrhythmic GABAergic chloride currents in MCs (Fig 11A, *lower panel*). Because of this reduced phasic GC inhibition, MC activity increased, but both MC spikes and STOs were relatively desynchronized (Fig 11B), and gamma oscillations were greatly impaired (compare Fig 11C with Fig 2B).

In contrast, when *W*_*MC*→*GC*_ was increased to 8 times the default value (from 1 to 8), GCs were strongly excited, spiking in response to many incoming EPSPs and maintaining a level of rhythmicity comparable to controls (Fig 11D, *upper panel*), but delivering much larger phasic GABAergic chloride conductances onto MCs (Fig 11D, *lower panel*). The increased level of phasic inhibition suppressed MC spikes, but retained the synchrony and periodicity of MC STOs (Fig 11E). Accordingly, a robust and coherent gamma oscillation persisted even with an 8-fold increase in the MC→GC synaptic weight, with little change in frequency (controls: 32.4 Hz; 800%*W*_*MC*→*GC*_: 35.4 Hz; Fig 11F).

**Fig 12:**
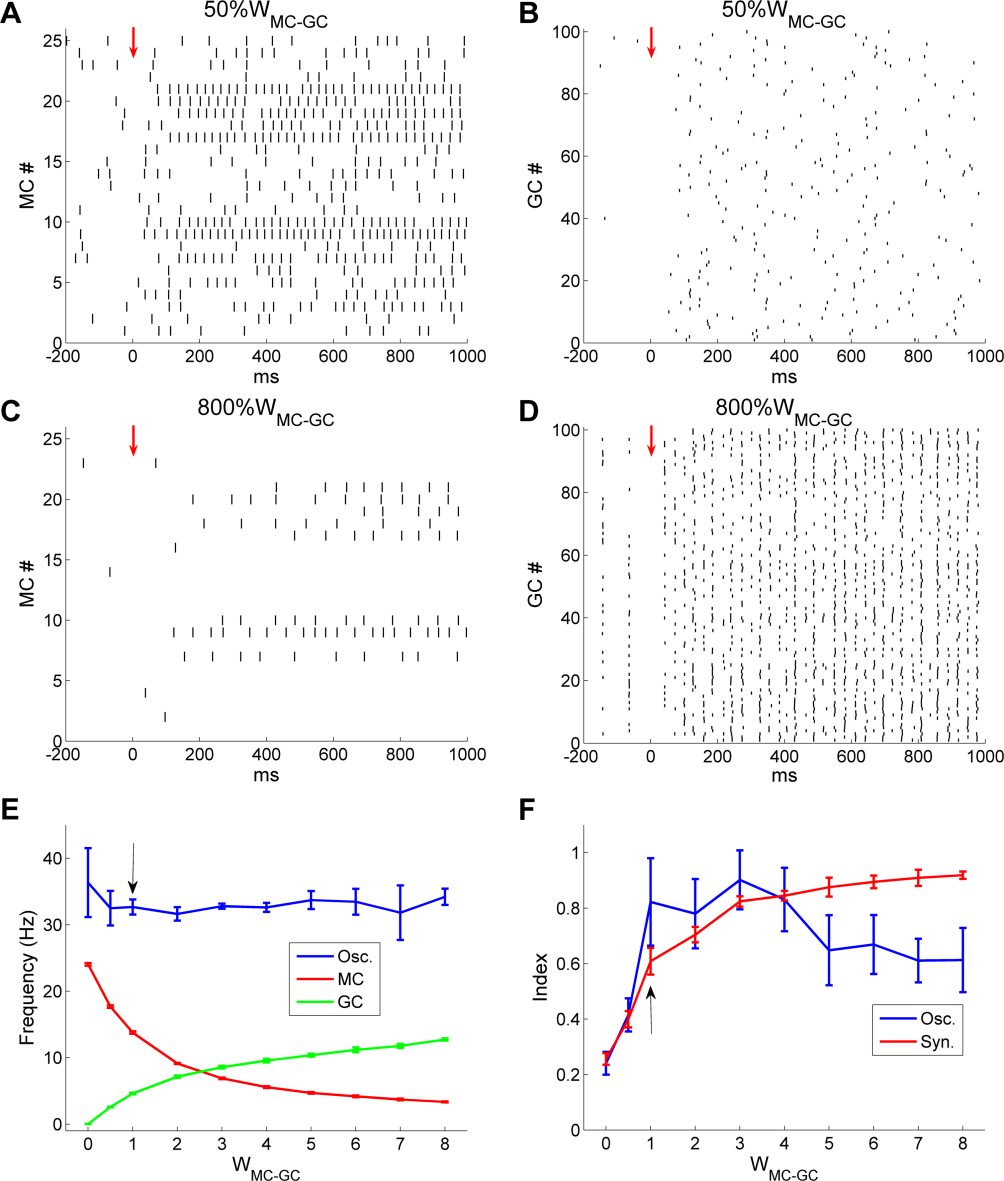
Gamma oscillations and spike synchronization persist under a wide range of MC→GC synaptic weights. ***A*:** Raster plot of MC spikes after MC→GC synaptic weights were reduced by 50% (50%*W*_MC-GC_). The *red arrow* designates the onset of odor input. ***B*:** Raster plot of GC spikes with 50%*W*_MC-GC_. ***C*:** Raster plot of MC spikes after MC→GC synaptic weights were increased eightfold (800%*W*_MC-GC_). ***D*:** Raster plot of GC spikes with 800%*W*_MC-GC_. ***E*:** Average odor-evoked MC and GC firing rates and sLFP oscillation frequency as functions of MC→GC synaptic weight. ***F*:** Synchronization and oscillation indices as functions of MC→GC synaptic weight. The default MC→GC synaptic weight was 1 (indicated by *black arrows* in ***E***, ***F***). Error bars denote standard deviations (SD).

To break this effect down further, we generated raster plots of MC and GC firing under these two conditions. When *W*_*MC*→*GC*_ was reduced by 50%, the mean odor-evoked GC firing rate was reduced from 4.6 Hz (in controls) to 2.6 Hz, resulting in a slight increase in the mean MC firing rate from 14 Hz (in controls) to 17.7 Hz. As noted above, network synchrony was reduced substantially (*SI*, controls: 0.63; *50%W*_*MC*→*GC*_: 0.40), because neither MC nor GC spike trains were well coordinated (Fig 12A and 12B). In contrast, with an eightfold increase in *W*_*MC*→*GC*_, GC firing rates were greatly increased (controls: 4.6 Hz; *800%W*_*MC*→*GC*_: 12.8 Hz) and GC spikes became remarkably well synchronized; this strong GC activation substantially suppressed MC firing (controls: 14 Hz; 800%*W*_*MC*→*GC*_: 3.3 Hz; Fig 12C and 12D). This substantially different balance of MC and GC activity was stable because one MC input was strong enough to produce correlated discharges in many postsynaptic GCs. Notably, under these conditions the mean MC firing rate (3.3 Hz across all MCs) was much lower than the oscillation frequency (35.4 Hz) and a majority of MCs exhibited no odor-evoked spikes (Fig 12C). The spikes of the remaining active MCs were effectively entrained by the highly synchronous GC activity, and exhibited elevated levels of synchrony (*SI*, controls: 0.63; *800%W*_*MC*→*GC*_: 0.92); i.e., coherent gamma oscillations persisted despite substantial increases in lateral excitatory synaptic weights. This is a particularly important stabilizing property given that the intrinsic OB synaptic plasticity underlying odor learning relies on the potentiation of excitatory synapses [47, 48].

The average odor-evoked MC/GC firing rates and sLFP oscillation frequencies across a range of MC→GC synaptic weights are depicted in Fig 12E. As *W*_*MC*→*GC*_ was increased, MC firing rates decreased while GC firing rates increased, eventually crossing. In contrast, the sLFP oscillation frequency remained stable (though unreliable at weights below 1 owing to very low spectral power; Fig 12E). The *OI* grew rapidly from its arrhythmic values at MC→GC synaptic weights below 1 up to a strong peak value that persisted across a fourfold range of excitatory synaptic weights, decreasing moderately thereafter (Fig 12F). This eventual decline arose as the increased activation of GCs began to impose tonic, as well as phasic, feedback inhibition that further reduced MC activation levels. In contrast, the *SI* increased steadily as *W*_*MC*→*GC*_ increased, gradually approaching unity at higher synaptic weights (Fig 12F).

### Gamma oscillations are robust to heterogeneity in afferent input intensity

Heterogeneity in population activity levels, whether across the neurons of an active ensemble or within a given population over time, poses a challenge to the stability and consistency of dynamical systems [60–65]. For example, the frequencies of gamma oscillations driven by pure PING mechanics vary directly with the activation levels of the excitatory neurons [58], which in the olfactory bulb are strongly heterogeneous (indeed, heterogeneity in MC activation levels is the fundamental basis of olfactory sensory representations). Notably, systems of coupled oscillators often are robust to reasonable heterogeneities in excitation levels [66]; indeed, the essence of coupled oscillator systems is a dynamics by which intrinsic differences in the natural frequencies of constituent oscillators are drawn together into a common limit cycle.

**Fig 13:**
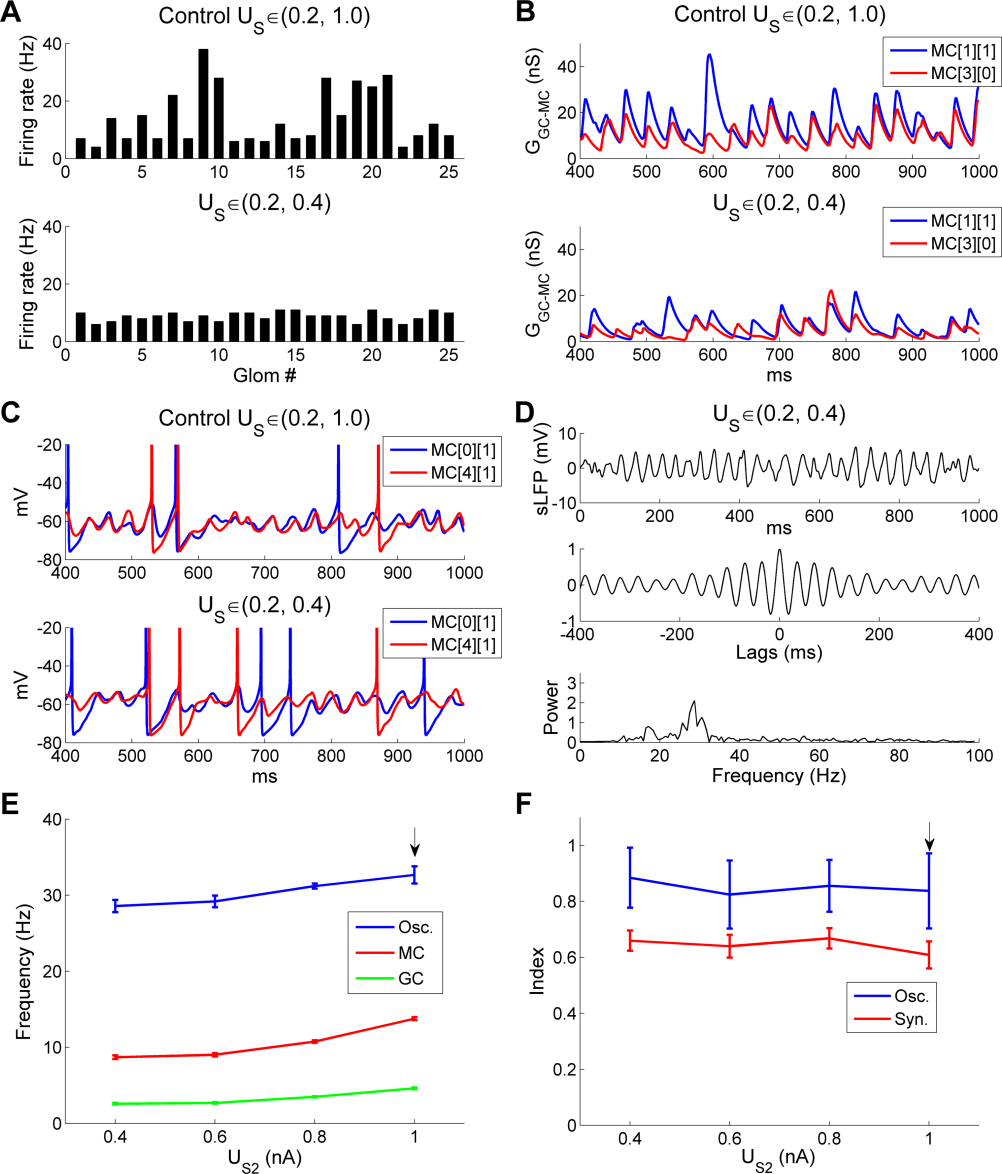
OB gamma oscillations are robust to variation in the steady-state upper bound of afferent input. ***A*:** Odor-evoked firing rates of all 25 MCs under control conditions (*top*) and following a reduction in the steady-state upper input bound (U_S2_) from 1.0 nA to 0.4 nA (*bottom*). ***B*:** Timeseries of cumulative GC-mediated GABA_A_ synaptic conductances in two representative MCs under control conditions (*top*) and following a reduction in the steady-state upper input bound from 1.0 nA to 0.4 nA (*bottom*). ***C*:** Membrane potential timeseries in two representative MCs under control conditions (*top*) and following a reduction in the steady-state upper input bound from 1.0 nA to 0.4 nA (*bottom*). ***D*:** Simulated LFP (*top*) during odor presentation, with autocorrelation (*middle*) and frequency power spectrum (*bottom*), after the steady-state upper input bound was lowered to 0.4 nA. ***E*:** Average odor-evoked MC and GC firing rates and sLFP oscillation frequency as functions of the steady-state upper bound of afferent input (U_S2_). ***F*:** Synchronization and oscillation indices as functions of the steady-state upper input bound (U_S2_). In these simulations, the steady-state lower input bound was maintained at its default (0.2 nA). The default upper input bound was 1.0 nA (indicated by black arrows in ***E***, ***F***). Error bars denote standard deviations (SD).

To assess the robustness of the OB network gamma oscillation to variance across the afferent input levels of MCs, we altered the ranges of excitation generated across the MC population by simulated odorant stimuli. By default, steady-state odor input intensities *u*_s_ (nA) were drawn from a uniform distribution within a bounded range (U_S1_, U_S2_). We first varied the upper input bound U_S2_ from 0.4 nA to 1.0 nA with increments of 0.2 nA, with the lower input bound U_S1_ fixed at 0.2 nA (Fig 13). When the upper input bound was reduced from 1.0 nA (in controls) to 0.4 nA, the odor-evoked MC firing rate dropped from 14 Hz to 8.8 Hz and the MC firing rate variance was markedly reduced (SD, *u*_*s*_ ∈ (0.2, 1.0): 9.8 Hz, *u*_*s*_ ∈ (0.2, 0.4): 1.6 Hz; Fig 13A). Because of the reduced MC drive, the odor-evoked GC firing rate also declined from 4.6 Hz to 2.4 Hz, and the reduction in GC excitation generated much smaller GABA_A_ conductance fluctuations on MCs (Fig 13B); this feedback response limited the overall change in the balance of excitation and inhibition. Despite these changes in firing rates and the amplitudes of synaptic interactions, MC oscillations remained highly synchronized under both conditions (Fig 13C), and the synchronization index was essentially unchanged (*SI*, *u*_*s*_ ∈ (0.2, 1.0): 0.63; *u*_*s*_ ∈ (0.2, 0.4): 0.62), and the frequency of the dominant sLFP spectral peak was only slightly reduced (*u*_*s*_ ∈ (0.2, 1.0): 32.4 Hz; *u*_*s*_ ∈ (0.2, 0.4): 28.7 Hz; compare Fig 13D with Fig 2B).

The mean odor-evoked neuronal firing rates and sLFP oscillation frequencies across a range of upper input bounds are depicted in Fig 13E. As the upper input bound increased from 0.4 nA to 1.0 nA, the mean MC firing rate increased 58.6% (from 8.7 Hz to 13.8 Hz) and that of GCs increased 76.9% (from 2.6 Hz to 4.6 Hz). In contrast, there was only a 14.3% increase in oscillation frequency (from 28.6 Hz to 32.7 Hz), demonstrating the relative robustness of OB gamma frequency to input variance. The synchronization and oscillation indices for the same range of upper input bounds are shown in Fig 13F. Both indices also demonstrated considerable stability in response to changes in the upper input bound.

**Fig 14:**
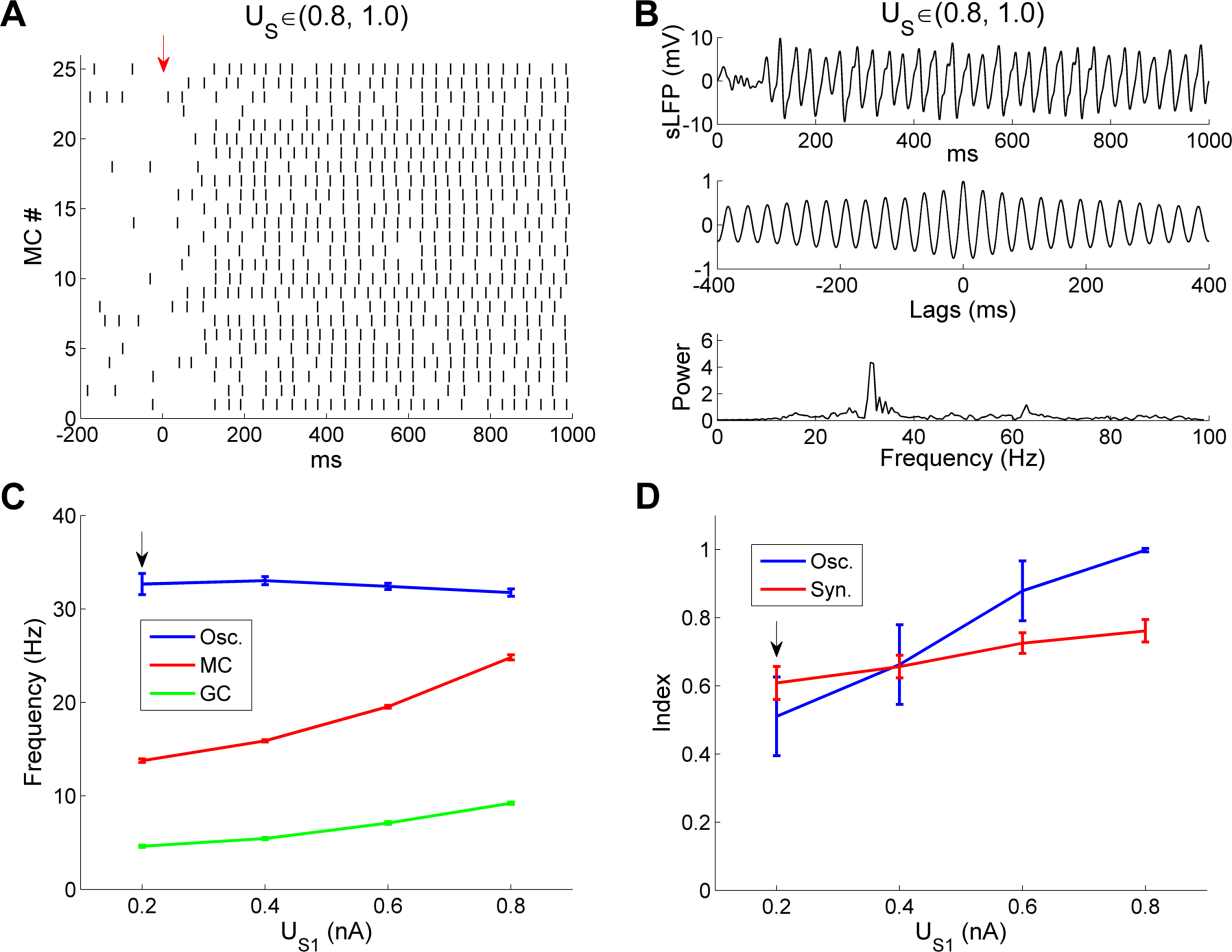
OB gamma oscillations are robust to variation in the steady-state lower bound of afferent input. ***A*:** Raster plot of MC spikes when the steady-state lower input bound (U_S1_) was increased fourfold, from 0.2 nA to 0.8 nA. The *red arrow* designates the onset of odor input. ***B*:** Simulated LFP (*top*) during odor presentation, with autocorrelation (*middle*) and frequency power spectrum (*bottom*), after the steady-state lower input bound (U_S1_) was increased to 0.8 nA. ***C*:** Average odor-evoked MC and GC firing rates and sLFP frequency as functions of the steady-state lower input bound (U_S1_). ***D*:** Synchronization and oscillation indices as functions of the steady-state lower input bound (U_S1_). In these simulations, the steady-state upper input bound was maintained at its default (1.0 nA). The default lower input bound was 0.2 nA (indicated by black arrows in ***C***, ***D***). Error bars denote standard deviations (SD).

We next fixed the upper input bound U_S2_ at 1.0 nA, and varied the lower input bound U_S1_ from 0.2 nA to 0.8 nA with increments of 0.2 nA (Fig 14). Increasing the lower input bound reduced input heterogeneity, as in Fig 13, but potentiated rather than reducing the average MC excitation level. When U_S1_ was increased from 0.2 nA to 0.8 nA, the odor-evoked MC firing rate increased from 14 Hz to 24.6 Hz, with markedly reduced variance (SD, *u*_*s*_ ∈ (0.2, 1.0): 9.8 Hz; *u*_*s*_ ∈ (0.8, 1.0): 3.6 Hz), leading to highly synchronized MC spikes (*SI*, *u*_*s*_ ∈ (0.2, 1.0): 0.63; *u*_*s*_ ∈ (0.8, 1.0): 0.7; Fig 14A and 14D). Accordingly, a strong, coherent sLFP gamma oscillation was generated with a higher-amplitude spectral peak than that exhibited by controls (Fig 14D; also compare Fig 14B with Fig 2B). However, despite this large increase in the mean MC firing rate, the sLFP oscillation frequency remained remarkably stable (*u*_*s*_ ∈ (0.2, 1.0): 32.4 Hz; *u*_*s*_ ∈ (0.8, 1.0): 31.1 Hz; Fig 14C).

### Glomerular-layer inhibition enables gamma oscillations by limiting MC excitation and firing rate heterogeneity

Coupled-oscillator networks are able to synchronize oscillators with nonuniform natural frequencies, but this robustness has limitations [61, 62, 66]. The large differences in input activation that can be generated by primary sensory receptor populations (responding to stimuli varying by orders of magnitude in physical intensity and receptive-field optimality) require regulation if they are to be constrained within the limited permissive range of the EPL’s oscillatory regime. Specifically, the range of absolute physiological variability generated in primary sensor populations must be compressed into a dynamic range that does not disrupt the functional dynamics of subsequent sensory system computations. This need is met in the early olfactory system by a series of concentration tolerance mechanisms (reviewed in [67]), culminating in a global normalization computation in the deep glomerular layer ([59]; corrected mechanism in [54]); this computation is mediated by the heterogeneous periglomerular/short-axon cell population [68, 69] and modeled herein by PGCs. To demonstrate the importance of these intensity compression mechanisms and examine the role of PGC-mediated inhibition in enabling OB gamma oscillations, we varied the PGC→MC synaptic weight (*W*_*PGC*→*MC*_) from 0 to 250% of its default value.

**Fig 15:**
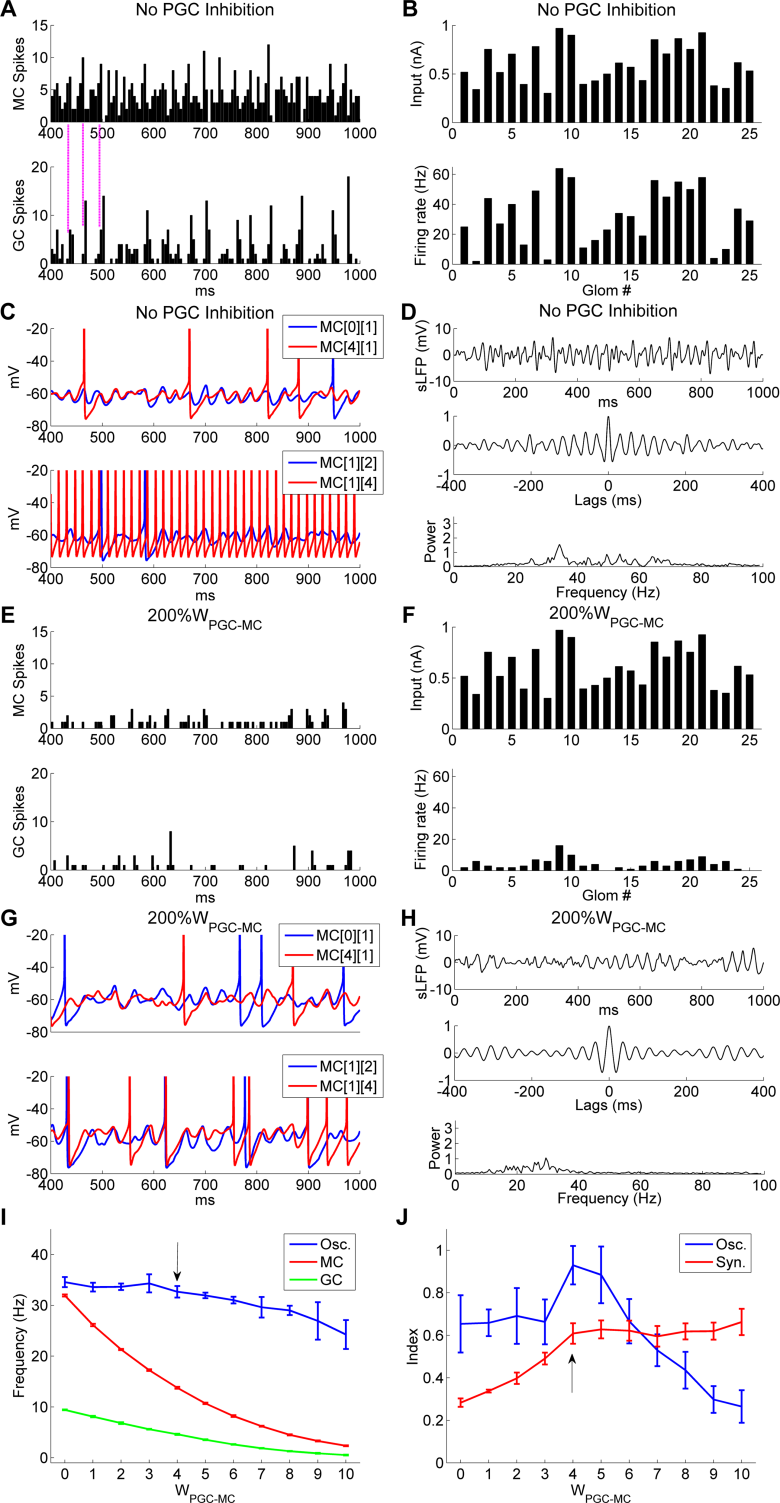
PGC inhibition enables OB gamma oscillations by limiting MC excitation and firing rate heterogeneity. ***A*:** Spike timing histograms of MCs (*top*) and GCs (*bottom*) without PGC inhibition (0%*W*_PGC-MC_). Vertical lines accentuate the alignment of spike time distributions. ***B*:** Steady-state OSN input intensities (*top*) and the odor-evoked firing rates of all 25 mitral cells (*bottom*) after PGC-mediated inhibition was blocked. ***C*:** Membrane potential timeseries of two example pairs of MCs during odor presentation in the absence of PGC inhibition. ***D*:** Simulated LFP (*top*) during odor presentation, with autocorrelation (*middle*) and frequency power spectrum (*bottom*), in the absence of PGC inhibition. ***E*:** As in ***A***, but with a twofold increase in PGC→MC synaptic weights (200%*W*_PGC-MC_). ***F*:** As in ***B***, but with a twofold increase in PGC→MC synaptic weights. ***G*:** As in ***C***, but with a twofold increase in PGC→MC synaptic weights. ***H*:** As in ***D***, but with a twofold increase in PGC→MC synaptic weights. ***I*:** Average odor-evoked MC and GC firing rates and sLFP oscillation frequency as functions of PGC→MC synaptic weight. ***J*:** Synchronization and oscillation indices as functions of PGC→MC synaptic weight. The default PGC→MC synaptic weight was 4 (indicated by black arrows in ***I***, ***J***). Error bars denote standard deviations (SD).

When PGC inhibition was entirely removed (*W*_*PGC*→*MC*_ = 0), the average odor-evoked MC firing rate increased markedly, from 14 Hz (in controls) to 32.2 Hz, inducing a concomitant increase in the mean GC firing rate (from 4.6 Hz to 9.5 Hz; compare Fig 15A with Fig 3D). Firing rates within the MC ensemble also displayed a much larger variance when PGC inhibition was removed (SD, controls: 9.8 Hz; No PGC inhibition: 19.3 Hz; compare Fig 15B with Fig 2A). Importantly, the removal of PGC inhibition significantly degraded MC spike synchrony (*SI*, controls: 0.64; No PGC inhibition: 0.39); this reduction in *SI* arose because of the substantial increase in asynchronous background or noisy spiking in MCs (compare Fig 15A, *upper panel*, with Fig 3D, *upper panel*). Nevertheless, GC population activity still retained a high level of rhythmicity comparable to controls (compare Fig 15A, *lower panel*, with Fig 3D, *lower panel*), and imposed strong phasic inhibition on MCs. Examination of MC and GC population activities indicates that GCs spiked only in response to peak MC spike rates (Fig 15A, *dashed vertical lines*), and the resulting phasic inhibition from GCs only partially suppressed MC spikes (i.e., MC spikes persisted during peak phasic inhibition), in contrast to the complete periodic suppression of MC spikes by GC inhibition in controls (compare Fig 15A with Fig 3D). Moreover, spike rates in the most strongly driven MCs exceeded the frequency of the underlying STOs, violating the restrictions of coupled oscillator-derived synchrony and consequently wholly desynchronizing with the remainder of the MC population (Fig 15C, *lower panel*; Fig 15J). Because of the loss of these highly-activated MCs from the synchronous population, the oscillatory power was considerably reduced in the absence of PGC inhibition (compare Fig 15D with Fig 2B), although a sizable spectral peak arising from the less-active MC population still persisted, exhibiting little change in frequency (controls: 32.4 Hz; *No PGC inhibition*: 34.2 Hz). This result supports two important points: First, although PGC inhibition improves global synchrony–specifically, it improves global participation in the synchronous ensemble by limiting the absolute activation levels of MCs to within a permissive range–it is not required for the generation of the OB gamma rhythm (Fig 15D), whereas GC inhibition is clearly required for OB gamma oscillogenesis (Fig 9B). Second, and critically, these results make clear that this coupled-oscillator mechanism is capable of sustaining coherent oscillations among participating MCs–i.e., those that are both within the permissive band of afferent activation levels and adequately coupled via MC/GC synaptic weights–irrespective of the additional presence of substantial numbers of active MCs that are non-participants in the coherent assembly (Fig 15C). As MCs are known for high levels of background spiking activity, both *in vitro* and *in vivo* but especially in awake/behaving animals [70], it is critical to determine the extent to which this activity is likely to interfere with the transmission of neural information. Experimental studies and theoretical models of gamma-timescale coincidence detection in the piriform cortex have suggested that such postsynaptic temporal selectivity will naturally exclude most uncorrelated background activity in MCs from affecting third-order neuronal representations of odor information [71, 72]. However, the present model is the first to demonstrate that timing-based odor representations in the OB can persist in the presence of high levels of uncorrelated background spiking.

Increased PGC inhibition also disrupted OB oscillations (Fig 15E–15H). When the PGC→MC synaptic weight was increased twofold (from 4 to 8), the average odor-evoked MC firing rate decreased from 14 Hz to 4.5 Hz (compare Fig 15F with Fig 2A), reducing the mean GC firing rate from 4.6 Hz to 1.3 Hz. Because of the paucity of activity under this tonic inhibitory suppression, the MC-GC feedback loop was functionally disrupted; GCs responded sparsely and weakly (compare Fig 15E with Fig 3D), evoking weak and irregular GABAergic synaptic conductances onto MCs. MC STOs thereby began to desynchronize and become irregular (compare Fig 15G to Fig 2C), and both gamma rhythm and power were seriously impaired (compare Fig 15H with Fig 2B).

Both the MC and GC mean firing rates decreased rapidly as *W*_*PGC*→*MC*_ increased further, whereas the sLFP oscillation frequency was stable below the control value and declined modestly at higher levels of PGC inhibition (Fig 15I), from 34.5 Hz at *W*_*PGC*→*MC*_ = 0 to 24.2 Hz at *W*_*PGC*→*MC*_ = 10. The synchronization index increased along with the strength of PGC inhibition up until the control value, and remained largely stable under stronger PGC→MC inhibitory weights (Fig 15J). In contrast, the oscillation index peaked around the control value and declined rapidly at higher PGC weights (Fig 15J). The discrepancy between *SI* and *OI* at large *W*_*PGC*→*MC*_ values arises largely from the fact that decreasing the numbers of spiking MCs does not reduce the *SI*, whereas the *OI* is sensitive to the desynchronization of driver currents and other subthreshold activity occurring among less strongly activated neurons. This highlights the fact that a correspondence between MC spikes and LFP deflections alone does not suffice to ensure coherent gamma oscillations.

These results show that PGC-mediated inhibition can serve to constrain the majority of MCs within a permissive range of activation. This constraint both protects the relational activation differences among MCs that underlie odor quality encoding and enables these odor-activated MCs to participate in a globally coherent gamma-oscillatory ensemble that constrains MC spike timing. Moreover, this globally coordinated oscillation, and the underlying phase-constraint of STOs and spikes in a majority of MCs, is robust to the potentially disruptive impact of highly active but uncorrelated MCs, whether uncorrelated owing to overstimulation or to inadequate coupling.

### Gamma oscillations are robust to network size

**Fig 16:**
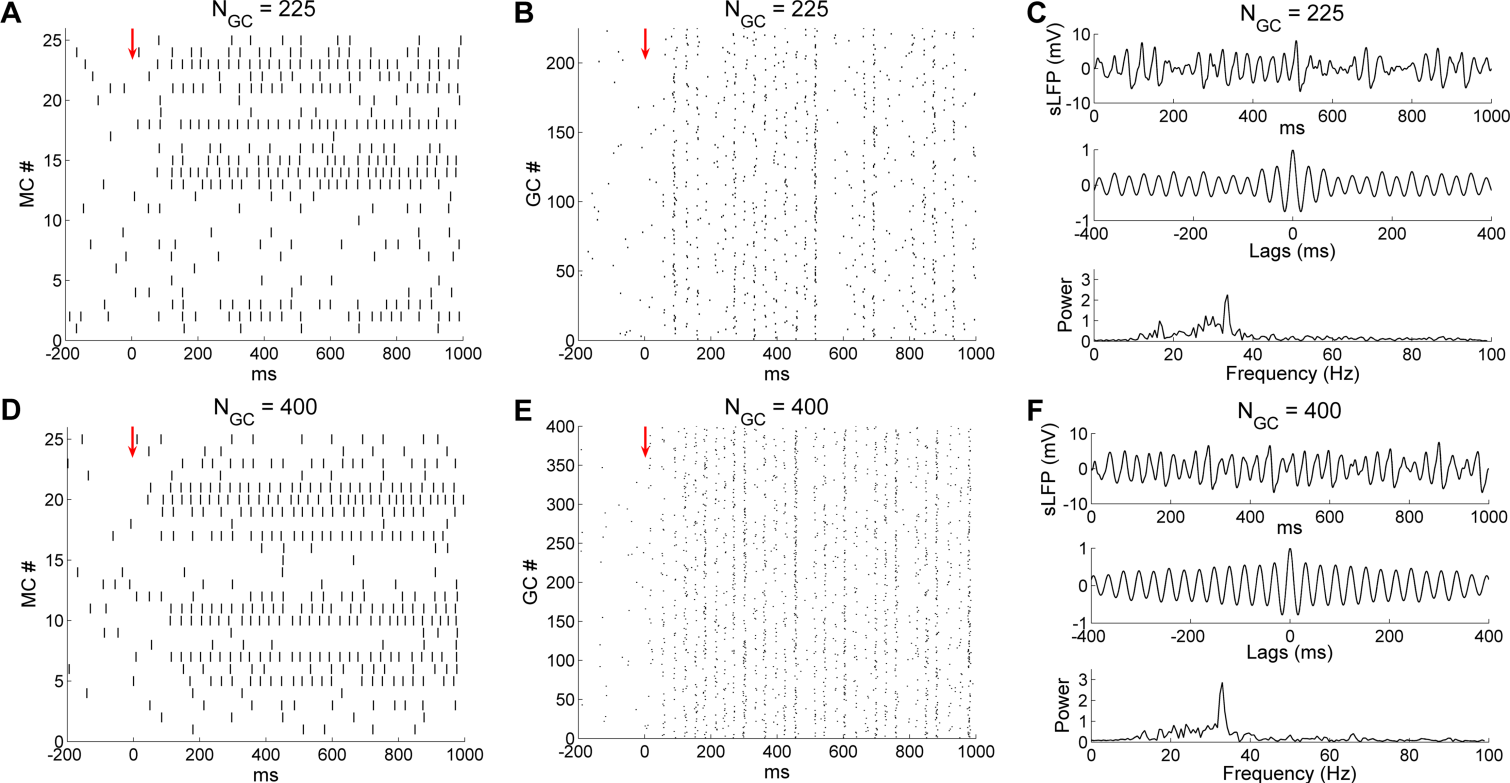
OB gamma oscillation is robust to variation in network size. ***A*:** Raster plot of MC spikes after increasing the number of granule cells in the model (N_GC_) to 225. The *red arrow* designates the onset of odor input. ***B*:** Raster plot of GC spikes with N_GC_ = 225. ***C*:** Simulated LFP (*top*) during odor presentation, with autocorrelation (*middle*) and frequency power spectrum (*bottom*), with N_GC_ = 225. ***D*:** Raster plot of MC spikes after increasing the number of granule cells (N_GC_) to 400. ***E*:** Raster plot of GC spikes with N_GC_ = 400. ***F*:** As in ***C***, but with N_GC_ = 400. The default network size was N_GC_ = 100.

Our OB network model contained 25 MCs, 25 PGCs and 100 GCs, a small fraction of the number of neurons in the biological OB; additionally, the ratio between the numbers of GCs and the numbers of MCs and PGCs is far greater than is represented in the model [1]. To test whether gamma oscillation in our model was robust to variations in this ratio, we increased the number of GCs (N_GC_) from 100 to 225 (15*15 array in Fig 1B) and 400 (20*20) respectively, while maintaining the number of MCs and PGCs at 25 each. To correct for the increased total inhibition that would be delivered onto MCs, we scaled down the maximal conductance of individual GC→MC synapses by the same factor such that the total GABA_A_ conductance received by each MC remained relatively constant. When N_GC_ was increased to 225, the mean odor-evoked MC and GC firing rates remained relatively unchanged (*controls*, MC: 14 Hz, GC: 4.6 Hz; *N*_*GC*_ *= 225*, MC: 13 Hz, GC: 4.2 Hz). Both MC and GC spikes displayed clear synchronization, and MCs displayed appropriately sparse spiking activity (Fig 16A and 16B). A dominant spectral peak in the sLFP power spectrum persisted at almost the same frequency and power as controls (*controls*: 32. 4 Hz; *N*_*GC*_ *= 225*: 33.6 Hz; compare Fig 16C with Fig 2B).

When N_GC_ was increased to 400, the mean odor-evoked MC and GC firing rates also remained stable (*controls*, MC: 14 Hz, GC: 4.6 Hz; *N*_*GC*_ *= 400*, MC: 14.2 Hz, GC: 5.2 Hz), and MC activity remained reasonably sparse (Fig 16D and 16E). A strong coherent gamma oscillation again persisted at approximately the same frequency and power as in controls (*controls*: 32.4 Hz; *N*_*GC*_ *= 400*: 33 Hz; compare Fig 16F with Fig 2B). While this variance does not encompass either the absolute size or the MC-GC ratio of the biological system, it does indicate that gamma oscillations are not highly sensitive to variations in network size.

## Discussion

The olfactory bulb transforms not only the information content of the primary sensory receptor input that it receives, but also its underlying coding metric. Large variance in absolute input amplitudes across receptor populations, varying on a slow respiratory timescale of encoding, are transformed by OB neural circuitry into patterns of ensemble spiking activity among OB principal neurons (mitral cells and projecting tufted cells) that are constrained in their amplitude variance and regulated on a fast gamma-band timescale. This emergent fast timescale for signaling is reflected in the gamma-band sLFP oscillations across the OB that are evoked by afferent activation of OB principal neurons, and presumably serves to efficiently integrate olfactory sensory information into the temporally regulated information networks of the central nervous system.

However, the physiological mechanism underlying this transformation has not been clear. Field potential oscillations at many frequencies are ubiquitous across the brain, and have been attributed to several different underlying dynamical frameworks. Each such theoretical framework imposes predictable relationships and limitations upon the activities of its constituent neurons, and defines the capacities and vulnerabilities of the network to changes in input statistics or internal parameter values. Multiple such frameworks–including PING, ING, STO-driven gamma oscillations, and the PRING hybrid mechanism described herein–have been proposed to underlie OB dynamics; among these, the PRING framework best corresponds to experimental observations of OB circuit neurophysiology [28, 35, 38]. The diagnostic elements of this PRING framework are (1) resonant principal neurons that receive external excitation (unpatterned on the gamma timescale) and exhibit intrinsic STOs, (2) reciprocal connectivity of these principal neurons with spiking inhibitory interneurons that do not separately receive afferent input, (3) a PING-like network oscillation that emerges under afferent activation; its frequency is determined principally by the decay time constant of the GABA(A) receptor conductance and must be higher than that of the STOs, thereby enabling a recurrent reset of STO phase in participating principal neurons, and (4) a continued dependence on principal neuron resonance properties during these network oscillations. In the present simulations, excitatory synapses were spike-mediated; inhibitory synapses were realistically graded but also compatible with GC spiking. Using a biophysically elaborated multiscale computational model of the OB, we here assessed the capacities and limitations of this PRING framework with respect to the observed properties of the OB circuit and the requirements of the olfactory sensory modality.

First, MCs converge onto piriform cortical pyramidal neurons from positions dispersed across the OB; there is no topographical organization to their projection patterns [73]. Coincidence detection in piriform pyramidal neurons [71, 72] requires that spike timing relationships among converging MCs be regulated by a common clock, so that incoming information is not dominated by random variance. Therefore, even physically distant MCs must be regulated by this common clock, indicating that EPL oscillations would need to be coherent across the entire layer, with negligible phase differences among regions. Such spatially extensive zero-phase coherent networks are nontrivial to construct, particularly in the presence of heterogeneous levels of activity among principal neurons. Coupled-oscillator networks in general, and our model here in particular, can yield robust coherence among excitatory neurons with negligible phase drift and across a wide range of physical scales, provided that there is sufficient direct long-distance synaptic coupling between distant columns (as provided here by the long MC lateral dendrites). When long-distance synaptic coupling is reduced in density, the spatial extent of coherence regions in the OB is correspondingly reduced [35], consistent with theoretical predictions [74–76].

Second, the mechanisms generating gamma oscillations should serve to phase-constrain informative MC spike timing, presumably with respect to a timescale appropriate for the synaptic integration time constants of postsynaptic follower neurons. Indeed, MC spikes are phase-constrained at the gamma/beta timescale [10, 13], and their follower neurons in piriform cortex exhibit key properties of coincidence detectors [71]. However, MCs also exhibit high levels of uninformative background spiking, and are particularly active in awake/behaving animals [70]. It is therefore equally important that the oscillogenic mechanism of the OB be robust to high levels of uncorrelated MC spiking. In our model, MC spikes are phase-constrained by virtue of intrinsic STO dynamics [28], which are periodically reset by GABA(A)-ergic synaptic inputs. The dynamical coordination and synchronization of these STOs and spikes across the full OB model is remarkably robust to the impact of high levels of uncoordinated MC spiking input (Fig 15; see also [77]). This robustness, together with the need for multiple convergent inputs to activate piriform pyramidal neurons [78], enables postsynaptic coincidence detectors to selectively respond to informative, temporally-coordinated MC inputs while disregarding MC background activity.

Third, this common frequency and zero-phase coherence must withstand substantial heterogeneity in afferent input levels, both across the network and over time. Heterogeneous networks are a challenge to synchronize [63–65], and, under many mechanisms, differentially-activated local regions of a heterogeneously-activated, spatially extensive network will exhibit different preferred frequencies [28, 33, 35]. Weak coupling has the capacity to pull such regions into a common oscillation, though it is generally effective only across a limited range of preferred frequencies and typically requires several, sometimes many, cycles to achieve synchronization [66, 79–81]. Stronger coupling, such as the STO phase-reset phenomenon of our coupled-oscillator model, enables a rapid, history-independent coordination among diverse local (columnar) oscillators across a range of activation levels [79]. The afferent activation-dependent differences among MCs in the rate of their recovery from synchronous GC-mediated synaptic inhibition have been proposed to generate the spike phase code exported from the OB [49, 72]; however, for present purposes, the important factor is that this coupling mode renders global sLFP synchronization robust to the large differences in afferent activation levels that together constitute the primary sensory representation (Figs 2, 13 and 14). Some dynamical frameworks also are not robust to inhibitory neurons that spike, or to networks in which excitatory or inhibitory neurons fire at dissimilar rates, or at rates far below the common oscillatory frequency. All of these phenomena are features of the OB network, and are robustly supported by the present model. Finally, global synchronization across the OB must also be robust to sparse network connectivity, and to substantial differences in synaptic weights across the EPL, particularly the excitatory synaptic weights that are modified during the process of odor learning [47, 48]. The present model maintains stable oscillations and global synchronization with sparse connections and a wide range of excitatory synaptic weights (Figs 11D–11F and 12).

Fourth, notwithstanding the above, there clearly are limits to the range of absolute input amplitudes that a dynamical system can withstand. The effects of afferent input intensity (concentration) are mitigated in animals by a series of compensatory mechanisms [67] capped by a global normalization network embedded in the OB glomerular layer, essentially feeding back a global average of input intensity as inhibition onto all MCs. This global normalization function was proposed a decade ago [51, 59], but the underlying circuit mechanism has only recently been determined [54]. In the model, as predicted, reduction of this circuit-based concentration tolerance by modifying PGC inhibition increased mean activity and variance across the MC population and disrupted spike synchronization (Figs 8 and 15).

PRING oscillations exhibit these diverse and computationally important properties by virtue of their integration of PING and STO mechanics. Two prior conductance-based network models of OB gamma oscillations also have incorporated both synaptic and STO dynamics [22, 33], but each reached different conclusions owing to differences in implementation. The earlier of these models, by Bathellier et al. [22], incorporated STO dynamics in single-compartment MCs, but did not include explicit GCs; instead, MC spikes directly generated recurrent and lateral inhibition, and there was no graded contribution to synaptic inhibition. In this model, the resonant properties of MCs were found to play little role in the gamma oscillation, and the population frequency depended on the rising time constant (rather than the decay time constant) of lateral synaptic inhibition. The second such model, by Brea et al. [33], incorporated explicit MCs and GCs, and exhibited both MC STO dynamics and graded synaptic inhibition. The Brea model demonstrated that STOs can be synchronized by graded inhibition, exhibited some STO resetting by this inhibition, and allowed mean MC firing rates to be much lower than the population oscillation frequency. However, it also differed from the present PRING model in several ways. First, in the Brea model, intrinsic STO frequencies directly drove the population oscillation frequency; the time constants of synaptic inhibition played little role. To accomplish this, MC STO frequencies were raised to 60–90 Hz, significantly higher than the 20–40 Hz that has been observed experimentally [28] and implemented in the present model. In principle, these high-frequency STOs could prevent the slower synaptic inhibition from determining the population frequency of the active network, as illustrated above (Fig 7E); however, in the Brea model, the STOs directly determined network frequency even when slowed to 35 Hz (Fig S5 in [33]). Differences in the properties of synaptic inhibition and GC spiking are more likely to be the main differentiating factors. Second, synaptic inhibition in the Brea model was activated at relatively hyperpolarized potentials (-66 mV), exhibited a relatively hard threshold (activated between -66.5 mV and -65.5 mV; Fig 1A of [33]), and was delivered directly to the single somatic compartment of the model cell. In contrast, in the present model, half-activation of the graded inhibitory synapses occurred at -40 mV, the threshold was much softer (activated between -50 mV and -30 mV), and incoming inhibitory synapses were distributed along an electrotonically extensive lateral dendrite. Third, the Brea model was not readily compatible with sparse GC spiking (i.e., GCs that spike at substantially lower frequencies than the population oscillation); in contrast, the present PRING model robustly supports sparse GC spiking during population oscillations.

In sum, the present model demonstrates that the PRING mechanism elucidated in the OB network by [35], when embedded in a multiscale, dynamical biophysical model of MC circuit function, exhibits the full set of dynamical properties that either have been experimentally demonstrated in the OB or are critical theoretical predictions based on experimental data. These experiments demonstrate that OB dynamics can be best described as independent columnar oscillators, coupled by pulsed inhibition, with a network topology based on long-distance, non-topographically organized connections. This elucidation of the essential dynamics of OB oscillogenesis will substantially constrain the plausible mechanistic hypotheses for interareal dynamics, such as the transient coherence in the beta band between OB and piriform cortex that characterizes particular phases of olfactory investigation.

## Methods

### The OB network model

The “default” OB network model contained 25 mitral cells (MCs), 25 periglomerular cells (PGCs) cells and 100 granule cells (GCs; [25]). Each MC, together with an associated PGC, represented a separate OB column, each of which was associated with a particular glomerulus and hence a distinct olfactory receptor type. The number of GCs in the model was increased substantially in certain simulations. The MC, PGC and GC single-cell models were Hodgkin-Huxley type conductance-based compartmental models based on those in [25]. In contrast to the 2013 model, the present OB network incorporated physical locations for each OB column in order to model the problems of distance-dependent lateral interactions, such as the differing propagation delays of spikes along MC lateral dendrites [50]. Specifically, the OB surface was modeled as a two-dimensional (2D) space (1 mm x 1 mm), upon which MCs and PGCs (together) and GCs (separately) were arranged in grid arrays with equal spacing in the horizontal and vertical directions (Fig 1B). To avoid edge effects, the 2D network was mapped onto a torus. Each neuron was labeled with its column and row numbers in the 2D space starting from 0 (i.e., MC[*i*][*j*] denoted the MC in the *i*th column and the *j*th row). In some figures, model neurons were denoted by a single index to enable their distribution along a single axis (e.g., in raster plots). In such cases, that single index *z* was related to the two indices *i* and *j* as follows: *z* = *N* * *i* + *j* + 1, where N was 5 for MCs and PGCs and 10 for GCs.

Both MC-PGC and MC-GC connections incorporated dendrodendritic synapses (Fig 1A; [25]). In the model, each MC formed reciprocal synapses with its local PGC (associated with the same glomerulus); i.e., the MC excited the PGC dendritic spine whereas the PGC inhibited the MC tuft compartment via graded inhibition (Fig 1A). MCs also interacted bidirectionally with GCs along the lengths of the MCs’ lateral (secondary) dendrites, which extend for long distances across the olfactory bulb [55, 82]. Specifically, MCs delivered synaptic excitation onto GC dendritic spines while receiving feedback and lateral inhibition from these same spines (Fig 1A). Each MC connected reciprocally to a random selection of GC dendrites with a connection probability *p* = 0.3. To model the cable effects of distance, the location of the dendrodendritic contact along the length of the seven-compartment MC lateral dendrite [25] was determined by the distance between the MC soma and the GC in question (Fig 1B).

### Synaptic currents

The MC→PGC and MC→GC synapses were mediated by both AMPA and NMDA receptors, whereas the PGC→MC and GC→MC synapses were mediated by GABA_A_ receptors. Postsynaptic currents were modeled as in [25]:

$$I_{syn}=Wg_{syn}sB(V)(V-E_{syn)}$$ (1)

where *g*_*syn*_ is the maximal synaptic conductance (prior to weighting) and *E*_syn_ is the reversal potential (0 mV for AMPA/NMDA currents and -80 mV for GABA_A_ currents; [25]). The maximum synaptic conductances were: *g*_*AMPA*_ = 2 nS and *g*_*NMDA*_ = 1 nS for both MC→PGC and MC→GC synapses, and *g*_*GABA*_ = 2 nS for both PGC→MC and GC→MC synapses [25]. *W* denotes the synaptic weight, which scaled the maximum preweighting synaptic conductance so as to generate final maximum synaptic conductances of W*g_*syn*_. The synaptic weight was varied systematically in simulations; default synaptic weights were: *W*_*MC*→*PGC*_ = 1, *W*_*MC*→*GC*_ = 1, *W*_*PGC*→*MC*_ = 4, and *W*_*GC*→*MC*_ = 2 (arbitrary units). The function *B*(*V*) implemented the Mg^2+^ block for the NMDA current, and was defined as *B*(*V*) = (1 + [*Mg*^2+^]exp(−0.062*V*)/3.57)^−1^ 83]. For AMPA and GABA_A_ currents, *B*(*V*) = 1. The gating variable *s* represented the fraction of open synaptic ion channels and obeyed first-order kinetics [84, 85]:

$$\frac{ds}{dt}=\alpha F(V_{pre})(1-s)-\beta s$$ (2)

where *F*(*V*_pre_) was an instantaneous sigmoidal function of the presynaptic membrane potential, *F*(*V*_*pre*_) = 1/(1 + exp(−(*V*_*pre*_ –*θ*_*syn*_)/*σ*)). The half-activation potential (*θ*_syn_) of the synapse was set to 0 mV for AMPA/NMDA receptor synapses and -40 mV for GABA_A_ synapses; the parameter σ was set to 0.2 for AMPA/NMDA currents and 2.0 for GABA_A_ currents [25]. Consequently, synaptic excitation was triggered mostly by spikes (high threshold), whereas synaptic inhibition occurred below spiking threshold and depended on presynaptic voltage in a graded manner. The channel opening rate constants (*α* and *β*) were expressed as *α* = 1/*τ*_*α*_ and *β* = 1/*τ*_*β*_, where *τ*_α_ and *τ*_β_ were the synaptic rise and decay time constants respectively. For AMPA receptor currents, *τ*_*α*_ = 1 ms, *τ*_*β*_ = 5.5 ms; for NMDA receptor currents, *τ*_*α*_ = 52 ms, *τ*_*β*_ = 343 ms; and for GABA_A_ receptor currents, *τ*_*α*_ = 1.25 ms, *τ*_*β*_ = 18 ms [25]. Such first-order synaptic models naturally simulate the interactions of successive presynaptic events, enabling the saturation of slow synapses [85]. Specifically, with a slow rising time constant of 52 ms, the NMDA conductance increased only slightly in response to a single presynaptic spike, but accumulated over multiple synaptic inputs owing to its slow decay time constant of 343 ms, limited by the maximum synaptic conductance. Different synaptic decay time constants have been reported by experimental studies in the OB [20, 23, 86]; importantly, the modeled time constants represent in part the “functional time constants” generated by a quasisynchronously activated population of presynaptic synapses affecting the same postsynaptic neuron. Some of these parameters were varied for purposes of particular simulations as described in the Results; in those cases, the parameter values specified above are referred to as “default” or “control” values.

### Specific and background inputs

Odor stimulation was modeled as in [25]. A sigmoidal function was used to model OSN inputs [33]:

$$I_{OSN}=u_{0}+0.5(u_{s}-u_{0})[\tanh\left(\frac{3(t-t_{ORN})}{r}-3\right)+1]$$ (3)

where *u*_0_ was the pre-odor value (simulated pure air input) and *u*_s_ the steady-state value after odor excitation. The parameter *r* determined the transition rate from *u*_0_ to *u*_s_ (set to 100) and *t*_orn_ the time of odor onset. Different MCs (representing separate, independently-tuned glomeruli) received different levels of afferent activation; the corresponding values of *u*_0_ and *u*_s_ were drawn from uniform distributions *u*_0_ ∈ (0.1, 0.2) and *u*_*s*_ ∈ (0.2, 1.0). Additionally, all cells in the network received random excitatory inputs representing intrinsic and extrinsic sources of uncorrelated background noise. These nonspecific inputs were modeled as uncorrelated Poisson spike trains mediated exclusively by AMPA receptors; specifically, they comprised instantaneous steps followed by exponential decays with a time constant of 5.5 ms [25]. When plotting major network measures (e.g., MC/GC firing rates, oscillation frequencies, synchronization and oscillation indices) under variable parameter sets, the data reported were averaged across 10 instantiations of the network with different random seeds for these Poisson spike trains.

### Simulated local field potential and spike phases

A simulated local field potential (sLFP) was constructed by filtering the mean (somatic) membrane potentials across all MCs [25]. Filtering was carried out numerically using a band-pass filter (10–100 Hz) with the MATLAB functions FIR1 and FILTFILT [33]. The power spectrum of the signal was obtained by a fast Fourier transform (FFT) of the filtered sLFP. MC somatic spike times were converted to spike phases using the method detailed in [25].

### Synchronization and oscillation indices

The synchronization (or phase-locking) index was calculated as follows [22]:

$$\kappa =1/N\sqrt{{\left[\sum_{i=1}^{N}sin(\varphi_{i})\right]}^{2}+{\left[\sum_{i=1}^{N}cos(\varphi_{i})\right]}^{2}}$$ (4)

where φ_i_ was the phase of each MC spike in the network relative to the sLFP peak. This synchronization index (*SI*) measures the degree of phase locking between MC spikes and sLFP oscillations rather than the absolute synchrony of MC spikes in time. Nevertheless, when substantial numbers of MC spikes are evoked, the *SI* also is a good measure of absolute spike synchrony. When all MC spikes have identical phases, the index achieves its maximal value of unity. The oscillation index (*OI*) corresponded to the peak of the frequency power spectrum of the sLFP, which was normalized to the largest peak value generated from ten sets of simulations with different random seeds. The oscillation frequency was determined from the position of the spectral peak in the power spectrum [25].

### Numerical methods

The model was implemented in the neuronal simulator package NEURON, version 7.3 [87], using the Crank-Nicholson integration method and a fixed timestep of 2 μsec (0.002 ms). Shorter timesteps did not change the results. Simulations were run both on a workstation under CentOS Linux and on Linux clusters provided by the Cornell Computational Biology Service Unit’s High Performance Computing laboratory (BioHPC). Simulation output data were saved in files and analyzed using custom Matlab scripts.
